# How to optimize the CAR-T Cell therapy process? A group concept mapping analysis of preconditions for a frictionless process from a German multistakeholder perspective

**DOI:** 10.3389/fonc.2024.1466803

**Published:** 2024-09-23

**Authors:** Ann-Cathrine Siefen, Melina Sophie Kurte, Florian Jakobs, Marcel Teichert, Bastian von Tresckow, Hans Christian Reinhardt, Udo Holtick, Johannes Atta, Christian Jehn, Elisa Sala, Anke Warnecke, Mathias Hänel, Christof Scheid, Florian Kron

**Affiliations:** ^1^ Faculty of Medicine, University of Duisburg-Essen, Essen, Germany; ^2^ VITIS Healthcare Group, Cologne, Germany; ^3^ Department of Hematology and Stem Cell Transplantation, Faculty of Medicine and University Hospital Essen, University of Duisburg-Essen, Essen, Germany; ^4^ Department of Hematology and Stem Cell Transplantation, West German Cancer Center and German Cancer Consortium (DKTK Partner Site Essen), University Hospital Essen, University of Duisburg-Essen, Essen, Germany; ^5^ Department I of Internal Medicine, Faculty of Medicine and University Hospital Cologne, University of Cologne, Cologne, Germany; ^6^ Department of Hematology, Giessen and Marburg University Hospital (UKGM), Giessen, Germany; ^7^ Department of Hematology/Oncology and Stem-cell Transplantation, St Georg, Asklepios Hamburg, Hamburg, Germany; ^8^ Department of Internal Medicine III, University Hospital of Ulm, Ulm, Germany; ^9^ Department of Hematology and Medical Oncology, Division for Stem Cell Transplantation and Cellular Therapy, University Hospital Göttingen, Göttingen, Germany; ^10^ Department of Internal Medicine III, Klinikum Chemnitz, Chemnitz, Germany; ^11^ Fachhochschule für Oekonomie & Management (FOM) University of Applied Sciences, Essen, Germany

**Keywords:** CAR-T, hematology, cluster map, mixed-methods, barriers, process optimization, group concept mapping

## Abstract

**Introduction:**

Treatment with chimeric antigen receptor T (CAR-T) cells involves a large number of interdisciplinary stakeholders and is associated with complex processes ranging from patient-specific production to follow-up care. Due to the complexity, maximum process optimization is required in order to avoid efficiency losses. This study aimed at systematically determining the preconditions for a frictionless flow of the CAR-T process by surveying the stakeholders involved.

**Methods:**

A Group Concept Mapping (GCM) analysis, a mixed-methods participatory research, was conducted. CAR-T experts from different professional backgrounds went through three steps: 1) Brainstorming relevant aspects (statements) for a frictionless process, 2) Sorting the collected statements based on their similarity, and 3) Rating the importance and feasibility of each statement. A cluster map reflecting the overarching topics was derived, and mean ratings per statement and cluster were calculated.

**Results:**

Overall, 20 CAR-T experts participated. A total of 80 statements were collected, resulting in a map of the following 10 clusters (mean importance/feasibility): Information for patients and physicians (4.16/3.77), Supportive network (4.03/3.53), Eligibility of patients (4.41/3.63), Evidence, transparency and communication (4.01/3.33), Paperwork (4.1/2.52), Interface with pharmaceutical manufacturer (4.03/2.85), Reimbursement (4.29/2.31), Quality Management (4.17/3.18), Infrastructure of CAR-T clinics (4.1/2.93), and Patient-oriented processes (4.46/3.32).

**Discussion:**

The 80 statements underlined the complex and manifold nature of the CAR-T treatment process. Our results reflect the first step in overcoming hurdles: identifying potential hurdles and required preconditions. Decision-makers and stakeholders can use the results to derive strategies and measures to further promote a frictionless process.

## 1 Introduction

The introduction of chimeric antigen receptor T (CAR-T) cell therapies has dramatically changed the once limited therapeutic landscape for hematologic malignancies such as acute lymphoblastic leukemia (ALL), diffuse large B-cell lymphoma (DLBCL), high grade B-cell lymphoma (HGBCL), follicular lymphoma (FL), primary mediastinal large B-cell lymphoma (PMBCL), mantle cell lymphoma (MCL), and multiple myeloma (MM) (1–6).

Chimeric antigen receptors are synthetically produced receptors engineered to be expressed on a patient’s T cells, thus also called “living drugs” (7). They identify specific target molecules on a tumor cell’s surface, leading to activation of the immune system and thus eliminating cancer cells. As the biological properties of the cells are changed in CAR-T cell products, they are classified as advanced therapy medicinal products (ATMPs) and are therefore subject to centralized approval by the European Union (8). In Europe, the first CAR-T cell products were approved in 2018. Six CAR-T cell therapies are currently approved by the European Medicines Agency (EMA): axicabtagene ciloleucel (6), brexucabtagene autoleucal (5), ciltacabtagene autoleucel (3), idecabtagene vicleucel (1), lisocabtagene maraleucel (2), and tisagenlecleucel (4).

However, market approval does not mean that the therapies are accessible to all potential patients. Access to cell therapies varies by country and region and depends on various factors, e.g. regulatory processes, market development, sufficient reimbursement, or patient identification procedures (9).

Clinical studies showed that hematologic patients diagnosed with e.g., DLBCL can substantially benefit from CAR-T cell therapies. Results of pivotal trials for third line therapy of DLBCL indicated overall response rates and complete response rates (CR) of 53% (CR 39%) for tisagenlecleucel, 73% (CR 53%) for lisocabtagene maraleucel, and 83% (CR 58%) for axicabtagene ciloleucel (10–12).

Since 2022 CAR-T cell therapies have been approved by EMA in second-line settings for large cell lymphomas [axicabtagene ciloleucel for DLBCL and HGBL (13), and lisocabtagene maraleucel for DLBCL, HGBL, PMBCL, and FL3B (14)] confirming improved clinical outcomes in high-risk populations with primary refractory disease or relapse within 12 months after frontline therapy (15, 16). Thus, clinical guidelines for DLBCL recommend CAR-T cell therapy as standard treatment for this population leading to a recent (January 2024) adaption of the therapy recommendation, now differentiating between “CAR-T eligible” and “CAR-T ineligible” patients (17, 18).

German cancer registry statistics reported 18,320 and 13,560 incident cases (absolute case numbers) in 2020 for non-Hodgkin lymphoma (NHL) and leukemia, respectively (19). The Joint Federal Committee estimated that 440-700 (B-cell NHL) (20), and 50-65 (B-cell ALL) (21) patients per year would be eligible for CAR-T cell therapy. However, between the first CAR-T approval in August 2018 and May 2020, 399 (aggressive B-cell NHL) and 97 (B-cell ALL) patients have been treated in German certified centers, respectively (22). Canales Albendea et al. assessed the access to CAR-T therapy of DLBCL patients in four European countries concluding that between 29% and 71% of eligible patients were not treated with CAR-T cell therapy (23). In their 2020 report, surveying German certified CAR-T centers, the German Society for Hematology and Medical Oncology (Deutsche Gesellschaft für Hämatologie und Medizinische Onkologie; DGHO) emphasizes organizational process hurdles potentially impeding to fully exploit the treatment’s potential (22). The innovative treatment option has therefore not yet reached its full potential, leaving behind the assumption of hurdles to the implementation of the CAR-T cell therapy process (in the following referred to as “CAR-T process”) (23).

The CAR-T process (in Germany) can be summarized into the following steps: Referral to a certified hospital, patient information and examinations, clarification of cost reimbursement in advance (optional), clarification of product availability, holding therapy (optional), apheresis and shipping to the pharmaceutical manufacturer for cell modification, bridging therapy (optional), lymphodepletion, administration of CAR-T cells, management of side effects (if necessary), follow-up care (7, 24). In Germany, CAR-T therapies must be performed by certified centers (25). Certification requirements include, e.g., criteria regarding infrastructure and organization, personnel, and experience and expertise.

Barriers to CAR-T cell therapy access have been studied in a number of studies [e.g., (23, 26–29)]. Currently, about 40 German hospitals have accomplished certification (30).

Hoffmann et al. described barriers to CAR-T therapy differentiating between patient-related barriers (e.g., travel to clinics, lacking caregiver support) and physician-related barriers (e.g., late referral, knowledge gaps) (29). Complex processes related to treatment with CAR-T cells are accompanied with practical hurdles for medical personnel, higher resource consumption due to inefficiencies, and impaired access for patients. In addition to the process itself, system-related factors such as the appropriate reimbursement and legal frameworks may further complicate the process.

In Germany, CAR-T therapy is currently performed in inpatient settings and is associated with extensive quality requirements. In the 2020 DGHO report most surveyed CAR-T centers confirmed insufficient reimbursement (22). However, the reimbursement of CAR-T therapy is dynamically evolving aiming to account for these insufficiencies. Since the latest changes for 2024, the reimbursement structure is composed as follows:

i. Hospital stays and related procedures, in general, are reimbursed by DRG flat fees per case (Diagnoses Related Groups). To date, there is no specific DRG for CAR-T treatment, thus cases are usually grouped according to the main diagnoses.
ii. In addition to the flat fees, the German inpatient reimbursement system includes extra fees for costly innovative treatments. Thus, hospitals receive extra fees remunerating the CAR-T product. Fees are individually negotiated between sickness funds and clinics.
iii. Costs caused by the apheresis have been defined as part of the drug, thus not reimbursed by statutory health insurance (SHI) (according to § 4 (14) Medicinal Products Act (Arzneimittelgesetz, AMG)). Pharmaceutical companies and clinics negotiate fees to cover apheresis costs.
iv. Since costs for structural requirements and additional efforts required are not covered by the DRG flat fee, another additional fee has been introduced in 2024. Its amount should be defined depending on the costs surpassing the DRG fee. Fees are individually negotiated between sickness funds and clinics.

In contrast to the already systematically recorded sequence of CAR-T process steps, there is currently a lack of identification of potential weaknesses within the process. These and the subsequent evaluation by stakeholders who are familiar with the process lead to a catalog of measures that can be used to optimize the overall process.

This study aimed at systematically determining preconditions for a frictionless flow of the CAR-T process and to evaluate these in terms of importance and feasibility by surveying the stakeholders involved.

## 2 Methods

### 2.1 Study setting

**Figure 1:**
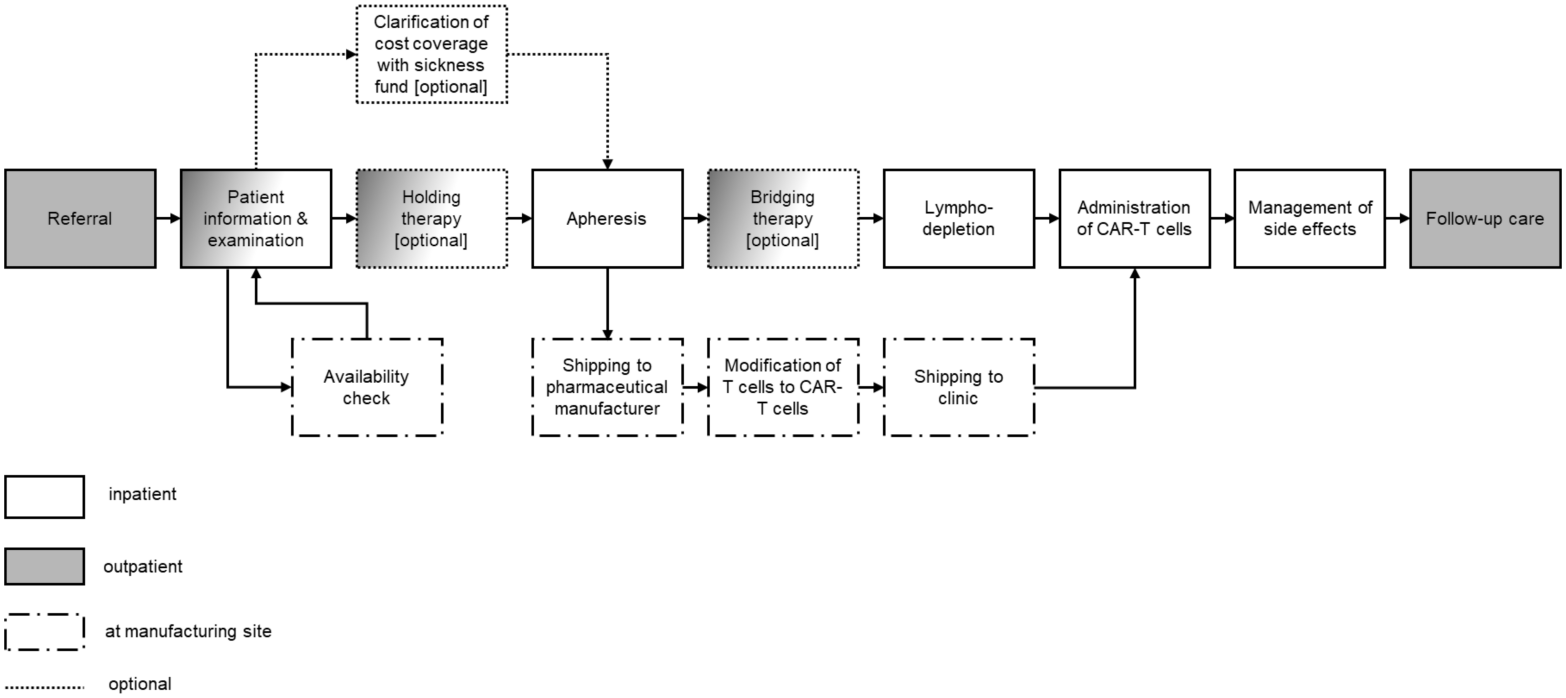
Process of CAR-T cell therapy. Own illustration based on Kron et al. (24) and clinical expert opinion.

The study was conducted from the viewpoint of the German healthcare setting (in- and outpatient) investigating the CAR-T process as depicted in **Figure 1**. Experts of different backgrounds (e.g. physicians, CAR-T coordinators, and health economists) involved in the CAR-T process were surveyed in 2023.

### 2.2 Study design

A Group Concept Mapping (GCM) Analysis was conducted to identify preconditions for a frictionless process of CAR-T therapy. GCM is “a structured methodology for organizing the ideas of a group or organization, to bring together diverse groups of stakeholders and help them rapidly form a common framework that can be used for planning, evaluation, or both” (31). As an integrative mixed-methods approach it combines qualitative and quantitative methods and results in various graphical depictions of the aggregated framework developed by the participants (31).

**Figure 2:**
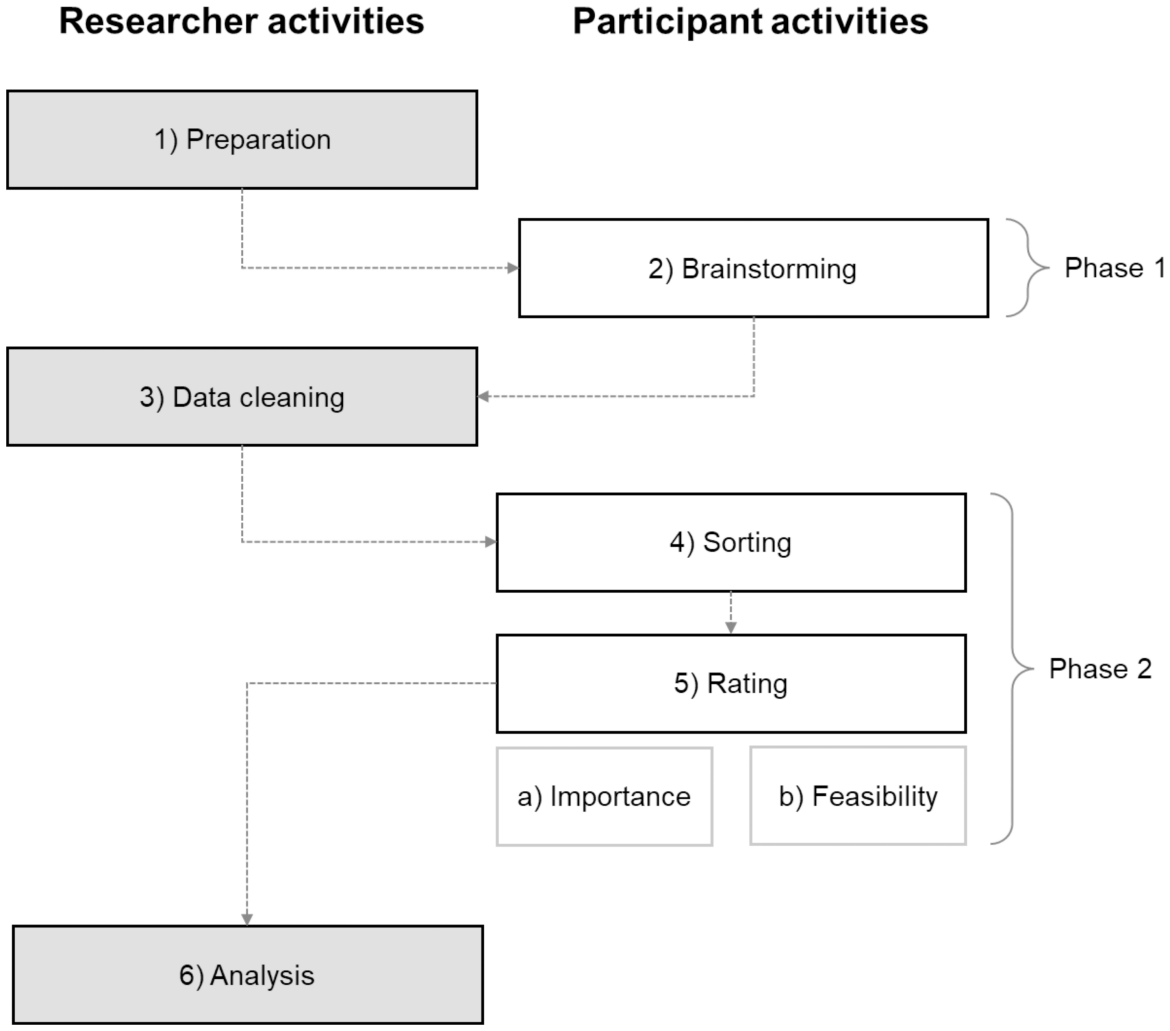
Group concept mapping process. Own illustration based on Kane and Trochim (31).

GCM analyses comprise six consecutive steps: 1) Preparation, 2) Brainstorming, 3) Data cleaning, 4) Sorting, 5) Rating, 6) Analysis. As a participatory research method, the underlying steps are divided into parts where participants are involved (2, 4, 5) and parts that are conducted by the researchers (1, 3, 6). **Figure 2** illustrates the GCM process.

Kane and Trochim developed a comprehensive manual describing GCM Analysis process in detail (31). The following paragraphs briefly describe the methodologic steps:

#### 2.2.1 Step 1: Preparation and selection of participants

The study’s research question was rephrased into a focus prompt based on a “finish the-sentence” format: “To ensure that the CAR-T cell therapy process - from initial indication to follow-up - runs as frictionless as possible, it takes…?”.

Next to the focus prompt, the rating dimensions – importance and feasibility – were specified. Purposive sampling was used to recruit participants with expertise related to the CAR-T process. Participant demographics that were to be collected were determined: professional groups, type of institution, years of CAR-T experience, number of CAR-T therapies per year in their institution.

#### 2.2.2 Step 2: Brainstorming

Brainstorming was conducted in two different manners. Purposively recruited participants either collected ideas in a remote meeting or on their own via the online platform groupwisdom™ (32). Brainstorming phase took place between June and September 2023. Participants were asked to collect ideas and topics of interest (statements) along the different parts of the CAR-T therapy process. Statements were collected in German and subsequently translated into English by the researchers.

#### 2.2.3 Step 3: Data cleaning

All collected statements were split into unique ideas, to ensure that each statement reflected only one aspect. Duplicates were eliminated and ambiguities and typos were removed. The resulting statement list formed the basis for the following steps.

#### 2.2.4 Step 4: Sorting

Participants were asked to group the final statements into piles based on the perceived similarity, i.e. similar statements were to be grouped together in one pile. The instructions were to sort all statements by similarity and to avoid creating piles for miscellaneous statements. Each pile was then to be labeled with a word or phrase describing the statements it contained.

#### 2.2.5 Step 5: Rating

Each participant rated each statement concerning its importance (‘How important is this aspect?’) and its feasibility (‘How easily can this aspect be implemented?’) separately. A 5-point Likert scale was used to indicate the importance/feasibility between “not at all” (1) and “extremely” (5). Steps 4 and 5 took place between September and November 2023.

#### 2.2.6 Step 6: Analysis

After all participants completed steps 1-5, the collected data were analyzed by the researchers using qualitative and quantitative methods. A variety of graphical and tabular results were generated with a visual concept map being the main result representing the ideas and their interrelationship.

##### 2.2.6.1 Step 6.1: Creating the cluster map

Data sorting formed the basis to generate a similarity matrix, indicating how often two statements had been assigned to one pile as a proxy for their similarity. By multidimensional scaling using the similarity matrix, a two-dimensional point map was generated. Statements that had been piled together frequently were located close to each other. Statements that had not (often) been piled together were located further apart on the point map. As the *exact* proximity of all statements cannot be depicted on two dimensions, it was necessary to diverge from the matrix. The degree of diversion was reflected in the so-called ‘stress value’ of a point map with lower values indicating a better fit. Stress values between 0.205 and 0.365 are considered as an indicator of a good fit (31).

Based on the generated point map, groups of points (i.e., statements) were used to build clusters (cluster analysis). One point map can lead to several versions of cluster maps, i.e. a different number of clusters can be possible. The researchers chose a final cluster solution by reviewing all cluster map solutions. Starting with the maximum number of clusters, the number of statements was continuously reduced until a comprehensive but compact version was determined. Afterwards, each cluster received a title based on titles given by the participants during the sorting task. The determination of the final cluster solution as well as the labeling of the clusters was performed jointly by two researchers (ACS and MSK).

To gain a comprehensive understanding of the final point map, a bridging analysis was performed: When placing the points on the map, similar statements, in general, are placed close to each other. If, however, a statement is similar to aspects on one side of the map, as well as to statements on the opposite side of the map, it is placed in-between. Such statements have higher bridging values (on a scale from 0 to 1) indicating higher heterogeneity. Statements with lower values are, in contrast, described as anchor statements reflecting a common idea of statements in their proximity.

##### 2.2.6.2 Step 6.2: Ratings

Average ratings of importance and feasibility (resulting from Step 5) were calculated per statement and per cluster. For a graphical depiction and comparison of the mean importance and feasibility ratings per cluster, a so-called Pattern Match graphic was generated. The Pattern Match contrasts mean ratings per cluster on vertical axes, one for each rating dimension.

##### 2.2.6.3 Step 6.3: Go-Zone

A so-called Go-Zone graphic is a two-dimensional map divided into four quadrants, in which the dividing lines reflect the mean importance and mean feasibility rating, respectively. All statements (displayed as points on this map) were located according to their mean importance and feasibility value. The Go-Zone reflects which statements were above or below the respective average. For instance, statements located in the upper right quadrant are perceived as more important and more feasible than average.

##### 2.2.6.4 Step 6.4: Subgroup analyses

To identify differences in the perceived importance and feasibility of less and more experienced participants, participants were split into two groups: Participants of centers administering CAR-T therapies below the yearly (case number) average were compared to those administering CAR-T therapies above the yearly average. Pattern Matches were generated to contrast the mean importance/feasibility rating of clusters per subgroups. This allowed a graphical depiction of the differences and similarities in the mean ratings between the subgroups.

### 2.3 Software

With exception of the brainstorming session conducted at the Advisory Board, all tasks were carried out remotely via groupwisdom™. The researchers used groupwisdom™ ^©^2022 (32) and Microsoft Excel for the analysis.

## 3 Results

### 3.1 Step 1: Preparation and selection of participants

In total, 20 experts took part in a minimum of one task. The experts were of different professions, including medical (50%), (health) economics (25%), pharmaceutical (15%) and medical assistant professions (5%). Most participants (60%) were working in qualified CAR-T cell therapy treatment centers). The entire expert group worked in Germany, thereof 40% in Federal States located in the west of Germany. Most experts (40%) reported 5 or more years of experience with CAR-T cell therapy with an average of 31 CAR-T cases treated per year. **Table 1** includes detailed participants’ characteristics.

**Table 1:** Participant characteristics per activity.

	Overall		Brain-storming		Sorting		Importance rating		Feasibility rating	
	n	%	n	%	n	%	n	%	n	%
Which professional group do you belong to?										
**Pharmaceutical**	3	15	3	17.65	3	18.75	3	17.65	3	18.75
**Medical**	10	50	8	47.06	7	43.75	8	47.06	7	43.75
**Nursing/medical assistant professions**	1	5	1	5.88	0	0	0	0	0	0
**(Health) economics**	5	25	4	23.53	5	31.25	5	29.41	5	31.25
**Other**	1	5	1	5.88	1	6.25	1	5.88	1	6.25
**Did not respond**	0	0	0	0	0	0	0	0	0	0
**TOTAL**	20	100	17	100	16	100	17	100	16	100
In which institution do you work?										
**Qualified CAR-T cell therapy treatment center (inpatient)**	12	60	10	58.82	10	62.5	10	58.82	9	56.25
**Referring clinic without CAR-T cell therapy (inpatient)**	1	5	1	5.88	1	6.25	1	5.88	1	6.25
**Outpatient institution in the hospital (MVZ, university outpatient clinic) of a qualified CAR-T center**	1	5	1	5.88	0	0	1	5.88	1	6.25
**Outpatient institution in the hospital (MVZ, university outpatient clinic) of a referring clinic**	0	0	0	0	0	0	0	0	0	0
**Outpatient (oncology) practice**	1	5	1	5.88	0	0	0	0	0	0
**Pharmaceutical company**	5	25	4	23.53	5	31.25	5	29.41	5	31.25
**Service sector**	0	0	0	0	0	0	0	0	0	0
**Other**	0	0	0	0	0	0	0	0	0	0
**Did not respond**	0	0	0	0	0	0	0	0	0	0
**TOTAL**	20	100	17	100	16	100	17	100	16	100
How many years of experience with CAR-T cell therapy do you have?										
**up to 2 years**	2	10	2	11.76	2	12.5	2	11.76	2	12.5
**over 2 to 3 years**	4	20	4	23.53	4	25	4	23.53	4	25
**over 3 to 4 years**	4	20	3	17.65	2	12.5	3	17.65	3	18.75
**over 4 to 5 years**	2	10	1	5.88	1	6.25	2	11.76	1	6.25
**over 5 years**	8	40	7	41.18	7	43.75	6	35.29	6	37.5
**Did not respond**	0	0	0	0	0	0	0	0	0	0
**TOTAL**	20	100	17	100	16	100	17	100	16	100
In which region of Germany is your institution located?										
**North (Bremen, Hamburg, Mecklenburg-Western Pomerania, Lower Saxony and Schleswig-Holstein)**	2	10	2	11.76	2	12.5	2	11.76	2	12.5
**East (Brandenburg, Berlin, Saxony, Saxony-Anhalt and Thuringia)**	3	15	2	11.76	2	12.5	3	17.65	2	12.5
**South (Bavaria and Baden-Württemberg)**	6	30	4	23.53	6	37.5	5	29.41	5	31.25
**West (North Rhine-Westphalia, Hesse, Rhineland-Palatinate and Saarland)**	8	40	8	47.06	5	31.25	6	35.29	6	37.5
**Did not respond**	1	5	1	5.88	1	6.25	1	5.88	1	6.25
**TOTAL**	20	100	17	100	16	100	17	100	16	100
How many CAR-T cell therapies are you/your institution involved in per year?										
**Minimum**	0		0		0		0		0	
**Maximum**	100		100		100		100		100	
**Mode**	40		40		40		40		40	
**Mean**	31		32		29.69		32.77		30.5	
**Count**	16		13		13		13		12	
**Median**	27.5		30		25		30		27.5	
**Variance**	689.63		699.85		680.67		734.64		728.92	
**Standard deviation**	26.26		26.45		26.09		27.10		27.00	
**Did not respond**	4		4		3		4		4	

### 3.2 Steps 2-3: Brainstorming and data cleaning

Seventeen participants took part in the brainstorming activity. Overall, 234 statements were collected from participants via Brainstorming (Step 2) which were split into 333 unique aspects by the authors. The final statement list comprised 80 statements reflecting major issues of interest (see **Table 2**) after 253 duplicates/redundant statements were removed in the data-cleaning procedure by the authors (Step 3). **Supplementary Table 1** encompasses the original statements and their English equivalent.

### 3.3 Steps 4-5: Sorting and rating

Sixteen participants contributed to the sorting of the statements. Seventeen participants rated the statements’ importance and 16 rated their feasibility. All sorting and rating data (Steps 4 and 5) were approved by the authors, i.e., none had to be excluded from the analysis.

### 3.4 Step 6: Analysis

#### 3.4.1 Step 6.1: Creating the cluster map

**Figure 3:**
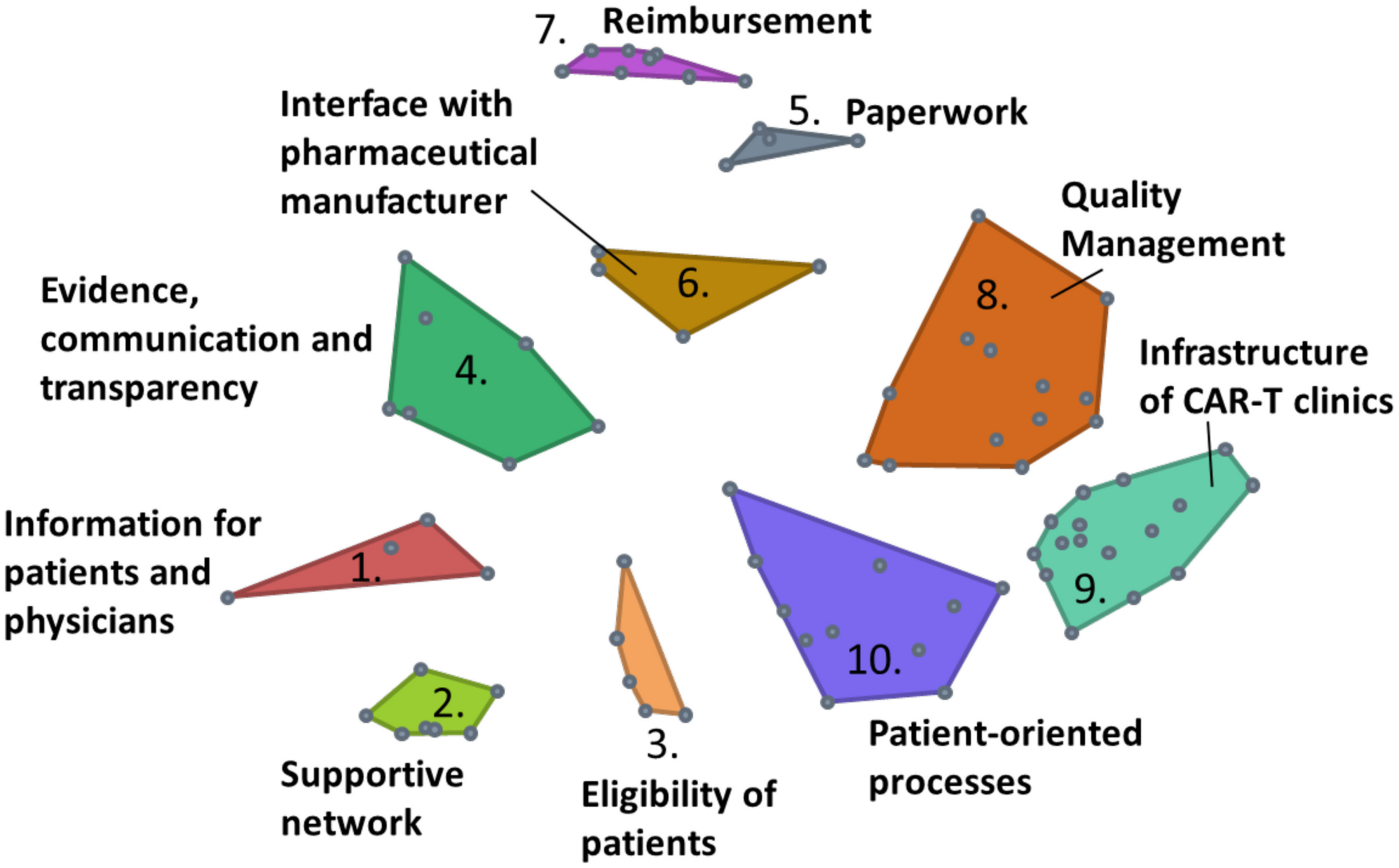
Cluster map (10-cluster solution). Each point represents one statement. Statements that have been piled together frequently are located closer to each other indicating similarity. Stress value = 0.2535.

The underlying point map has a stress value of 0.2535, indicating a good fit. Twelve cluster map options with 4 to 15 clusters were reviewed (see **Supplementary Table 2** for detailed information about each option). Finally, a map of 10 clusters was chosen, comprising clusters with the following titles: C1) *Information for patients and physicians*, C2) *Supportive network*, C3) *Eligibility of patients*, C4) *Evidence, communication and transparency*, C5) *Paperwork*, C6) *Interface with pharmaceutical manufacturer*, C7) *Reimbursement*, C8) *Quality Management*, C9) *Infrastructure of CAR-T clinics*, and C10) *Patient-oriented processes* (see **Figure 3**).

Clusters comprise between 4 (Cluster 1 *Information for patients and physicians* and Cluster 5 *Paperwork*) and 16 (Cluster 9 *Infrastructure of CAR-T clinics*) statements. **Table 2** summarizes all clusters and the associated statements. Average bridging values per cluster ranged between 0.08 (Cluster 7 *Reimbursement*) and 0.78 (Cluster 4 *Evidence, communication and transparency*). The two statements with the highest bridging values were *Contractually regulated collaboration (1.0)*, and *Bags with sufficient volume (0.95).* **Supplementary Table 3** shows the bridging values of all statements and clusters.

**Table 2:** Statements per cluster incl. mean ratings & quadrants.

			Importance	Feasibility	Quadrant on Go-Zone
Statement ID	Statement	N	mean	mean	
**Cluster 1**	**Information for patients and physicians**	4	4.16	3.77	
1	Target group and product-specific information (for referring physicians, patients, practitioners, nursing professionals, etc.)		4	3.8125	upper left
25	Shared decision making		4	3.875	upper left
31	Consideration of differences in real-world populations vs. study populations		4.117647	3.4375	upper left
49	Knowledge of and focused information on the possible spectrum of side effects during the course of the disease		4.529412	3.9375	upper right
**Cluster 2**	**Supportive network**	7	4.03	3.53	
14	Cancer survivorship measures		3.882353	3.1875	upper left
15	Social support services		3.941176	3.0625	lower left
17	Support for patients in the pre-treatment phase		4.176471	3.4375	upper left
21	A patient passport		3.529412	4.375	upper left
24	Patients´ health literacy		4.117647	2.9375	lower left
28	Support from the patient´s private/family environment		4.352941	3.625	upper right
48	Involvement of relatives in the explanation of medical issues		4.235294	4.0625	upper right
**Cluster 3**	**Eligibility of patients**	5	4.41	3.63	
10	Patients in overall good state of health (“fit”)		4.117647	2.625	lower left
11	Easily applicable risk scores to enable a differentiated approach in both outpatient and inpatient settings		4.235294	3.3125	upper right
56	Rapid clarification of the patient’s suitability for CAR-T therapy		4.705882	3.8125	upper right
62	Information on cytostatic drugs that can affect apheresis		4.352941	4.125	upper right
63	Individual information for patients		4.647059	4.25	upper right
**Cluster 4**	**Evidence, communication and transparency**	7	4.01	3.33	
7	Guidelines regarding specific cancers (e.g. DLBCL, CLL)		4.294118	3.5	upper right
8	Bags with sufficient volume		3.5	3.133333	upper left
18	Outcome data collection during the course of therapy and aftercare		4.352941	3.1875	upper right
22	Regular information on research projects and study participation for patient recruitment		3.941176	4	upper left
67	Transparency concerning out-of-specification (OOS)		4.117647	3.3125	upper left
74	Contractually regulated collaboration		3.823529	2.75	lower left
80	A clear definition for second-line therapy		4.058824	3.4375	upper left
**Cluster 5**	**Paperwork**	4	4.1	2.52	
3	Juristically secured cancellation policy		3.941176	2.875	lower left
9	Managing issues related to the Health Insurance Medical Service (Medizinischer Dienst; MD)		3.764706	2.625	lower left
59	Reduction of the bureaucratic burden		4.647059	2.25	lower right
65	Inclusion of apheresis in the joint federal committee (German: Gemeinsamer Bundesausschuss; G-BA) guideline		4.0625	2.3125	lower left
**Cluster 6**	**Interface with pharmaceutical manufacturer**	4	4.03	2.85	
50	Outpatient specialist care (German: ambulante spezialfachärztliche Versorgung - ASV) for hemato-oncologists (§116b Social Codebook V)		3.882353	3	lower left
61	Cross-industry standards		4.117647	2.266667	lower left
64	User-friendly ordering portal		4.352941	3.625	upper right
66	Manufacturing capacities in Europe		3.764706	2.5	lower left
**Cluster 7**	**Reimbursement**	9	4.29	2.31	
19	Legal certainty to avoid recourses by the Health Insurance Medical Service (Medizinischer Dienst; MD) and the associated certainty of reimbursement		4.529412	2.0625	lower right
23	More transparency in the development of reimbursement amounts following the AMNOG procedure		4.176471	2.875	lower left
32	Sector-specific, standardized coding rules		4	2.625	lower left
34	Specific DRG(s) for CAR-T cell therapy		4.235294	2.125	lower right
35	Sufficient and uniform nationwide refinancing (additional NUB fee)		4.705882	2	lower right
36	Lump sum for establishment and maintenance costs		4	2.1875	lower left
37	Adequate financing across sectors		4.705882	2.1875	lower right
51	Selective contractual agreements (§ 140a Social Codebook V)		3.529412	2.6875	lower left
54	Security of cost coverage in all sectors		4.764706	2.0625	lower right
**Cluster 8**	**Quality Management**	13	4.17	3.18	
2	Use of digital solutions such as telemedicine, electronic patient records and apps		3.647059	2.5	lower left
6	Anticipation of and investment in future developments (e.g. growth)		3.941176	2.875	lower left
29	Standardized measures and materials for documentation		4	3.5625	upper left
30	Good project management and standard operating procedures (SOP)		4.294118	3.875	upper right
33	Quality assurance and management		4.588235	3.25	upper right
38	Exchange of experiences		4.470588	3.875	upper right
46	Minimum possible time between completion of the product and product delivery		4.529412	2.75	lower right
55	Collaborative tumor boards		4.352941	4	upper right
69	Transparent, digital process tracking		4	3	lower left
70	Short vein-to-vein time		4.529412	2.75	lower right
71	Digitization of the process		3.941176	2.4375	lower left
72	Further training for personnel involved in the process		4.647059	3.625	upper right
76	Quality management of the product by the pharmacy		3.235294	2.875	lower left
**Cluster 9**	**Infrastructure of CAR-T clinics**	16	4.1	2.93	
4	Sufficient qualified personnel/personnel capacities		4.705882	2.0625	lower right
16	Provision of capacities for (long-term) side effect management		3.882353	2.8125	lower left
20	Few changes in “aftercare personnel”		3.764706	2.1875	lower left
26	Bed availability on normal ward		4.058824	2.75	lower left
27	Bed availability on intensive care unit		4.294118	2.5625	lower right
39	Good cooperation and communication within the institutions		4.588235	3.625	upper right
40	Good involvement of nursing personnel		4.705882	3.5	upper right
41	Coordinated discharge management		4.235294	3.5625	upper right
44	CAR-T cell coordinators/guides		4.647059	3.375	upper right
53	Specialized CAR-T centers		4.588235	3.4375	upper right
57	In-house apheresis units/apheresis networks		3.764706	2.75	lower left
60	Sufficient apheresis capacities		4.764706	2.625	lower right
68	In-house cryopreservation		3.176471	2.6875	lower left
77	Interdisciplinary teams		4.470588	3.5	upper right
78	Delivery to the cell laboratory by pharmacy		2.588235	2.9375	lower left
79	Stocking in the pharmacy		3.294118	2.5625	lower left
**Cluster 10**	**Patient-oriented processes**	11	4.46	3.32	
5	Clearly defined contact persons/contact options for all persons concerned (including patients)		4.823529	3.6875	upper right
12	Structured, cross-sector aftercare programs		4.470588	3.0625	lower right
13	Timely diagnostics		4.764706	3.1875	upper right
42	Good connection/access to the CAR-T center		4.705882	3.3125	upper right
43	Good integration of referring physicians		4.705882	3.3125	upper right
45	Bridging the time through appropriate bridging therapies		4.411765	3.6875	upper right
47	Avoidance of infections that would delay the process		4.411765	2.8125	lower right
52	Checklists for indications and preliminary medical examination		4.352941	4	upper right
58	Good cooperation and communication between the various institutions and professions		4.588235	3.375	upper right
73	Outpatient treatment, if possible (e.g. also outpatient lymphatic depletion)		3.588235	2.5	lower left
75	Efficient communication regarding changes to the timeline through bridging therapy		4.235294	3.625	upper right

#### 3.4.2 Step 6.2: Ratings

For average ratings of individual statements, values ranged between 2.59 (*Delivery to the cell laboratory by pharmacy*) and 4.82 (*Clearly defined contact persons/contact options for all persons concerned (including patients)*) for importance and between 2.0 [*Sufficient and uniform nationwide refinancing (additional NUB^1^ fee)*] and 4.37 (*A patient passport*) for feasibility. The average importance and feasibility across all statements is 4.19 and 3.1, respectively.

**Figure 4:**
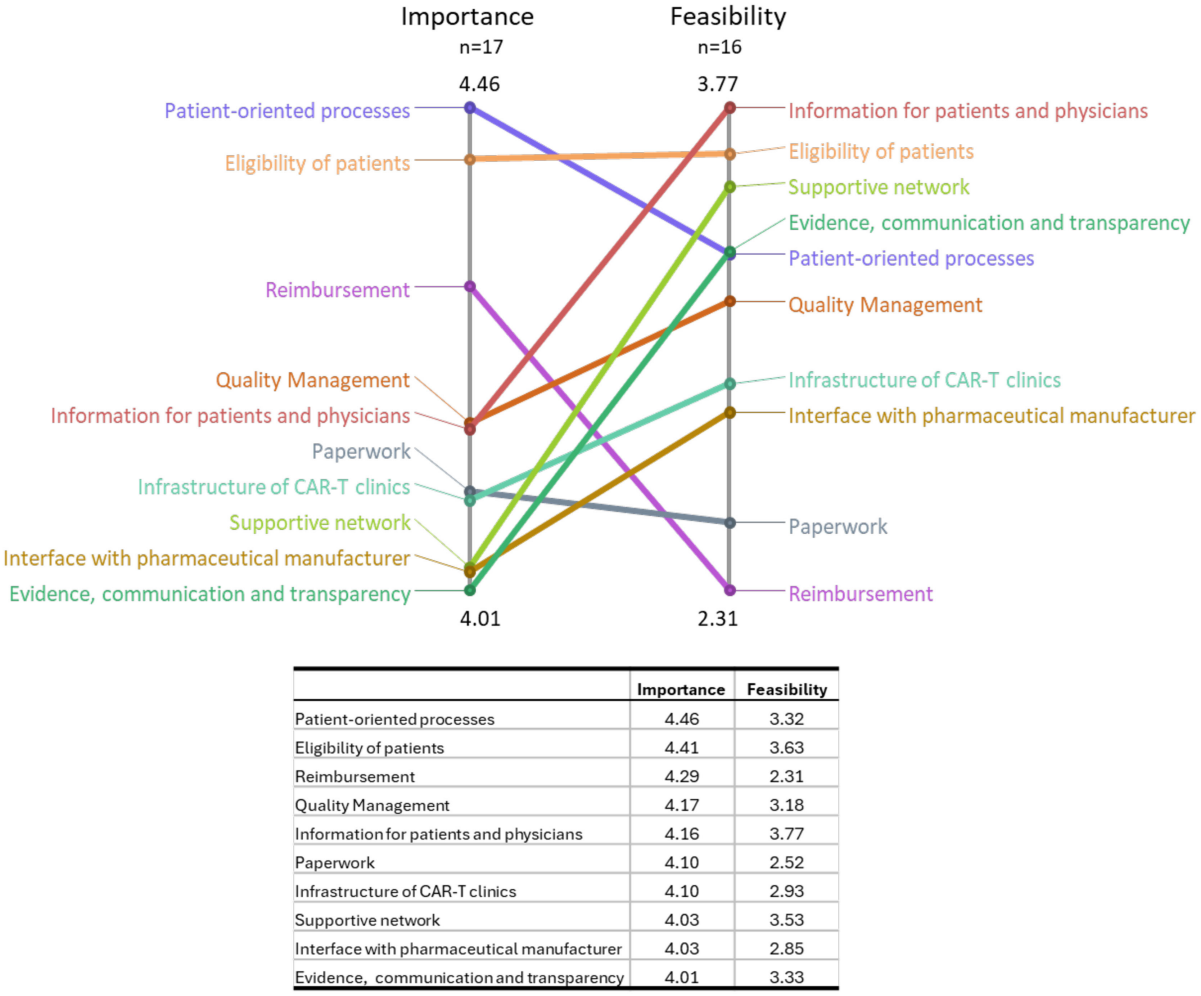
Pattern Match - Importance versus feasibility rating. Average importance and feasibility rating per cluster are contrasted. Axes values are *relative*, i.e., minimum and maximum values per dimension (not overall) are reflected. Ratings were done on a 5-point Likert scale ranging from 1 to 5.

On a cluster level, Cluster 10 *Patient-oriented processes* received the highest average importance value (4.46) and Cluster 1 *Information for patients and physicians* received the highest average feasibility rating (3.77). Lowest average importance and feasibility was perceived for Cluster 4 *Evidence, communication and transparency* (4.01) and Cluster 7 *Reimbursement* (2.31), respectively. Mean cluster ratings for importance and feasibility were contrasted in a Pattern Match in **Figure 4**.

#### 3.4.3 Step 6.3: Go-Zone

**Figure 5:**
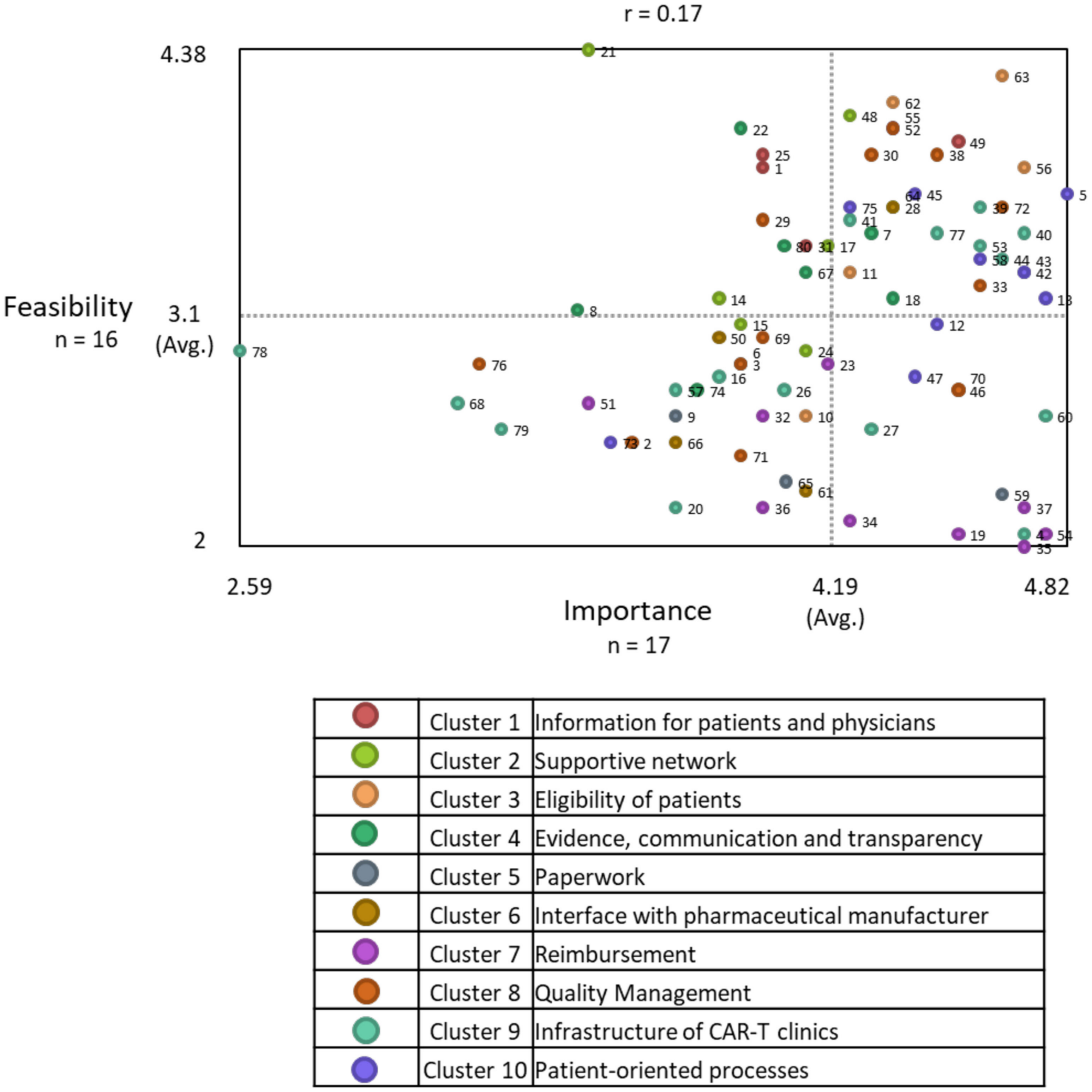
Go-Zone. The map is divided into four quadrants based on the average importance and feasibility rating across all statements. Statements are plotted on the graph depending on their average rating values. Each point reflects one statement. Their color indicates the associated cluster. For example, statements located in the upper right quadrant are perceived above average important and feasible.

**Figure 5** shows the resulting Go-Zone graphic. Division lines were drawn at 4.19 (importance axis) and 3.1 (feasibility axis). 42/80 statements (52.5%) were rated above-average important, and 40/80 statements (50%) were rated above-average feasible.

29/80 (36.25%) statements were in the upper right quadrant (important and feasible), and 13/80 (16.25%) statements were located in the lower right quadrant (important, less feasible).

Most statements in the upper right quadrant belong to Cluster 10 *Patient-oriented processes* (8/29; 27.59%) and statements in the lower right quadrant predominantly belong to Cluster 7 *Reimbursement* (5/13; 38.46%). **Supplementary Table 4** shows all statements per quadrant.

#### 3.4.4 Step 6.4: Subgroup analyses

**Supplementary Figure 1** shows the Pattern Match comparing feasibility results for above (n=6) and below (n=7) average CAR-T cases per year. It revealed that the following clusters were perceived less feasible by participants involved in more cases per year (>30 cases vs. <30 cases): *Interface with pharmaceutical manufacturer* (2.93 vs. 2.6), *Reimbursement* (2.29 vs. 2.11), and *Infrastructure of CAR-T clinics* (2.87 vs. 2.80). *Eligibility of patients* showed the same average rating (3.6) for both subgroups. Remaining clusters were perceived more feasible with higher case numbers. The comparison of mean importance ratings per subgroup showed lower importance ratings from the <*30 cases* subgroup for all clusters except Cluster 1 *Information for patients and physicians* and Cluster 6 *Interface with pharmaceutical manufacture*. However, the ranking of cluster importance was somewhat comparable (**Supplementary Figure 2**).

## 4 Discussion

The CAR-T process has been described as complex and hurdle-containing in various publications (23, 28, 33, 34). The first important step towards the establishment of a frictionless CAR-T process is the systematical process assessment in terms of potential hurdles and efficiency losses. Our analysis not only identified which topics are relevant but also assigned importance and feasibility values to them by surveying experts in Germany.

As a main finding, this study showed that patient-oriented processes, eligibility of patients and reimbursement are the three most important preconditions for a frictionless CAR-T process according to the surveyed experts. However, the analysis also showed that importance and feasibility of clusters are often discrepant. This is especially crucial for highly important topics that are perceived as poorly feasible, such as patient-oriented processes and reimbursement and special efforts must be made to comply with their importance.

The identified clusters can be categorized into provider-related, patient-related, and system-related factors. This is in line with previous studies, such as Hoffmann et al., who differentiated between patient-specific and physician-specific barriers for referral to CAR-T therapy or Gajra et al., who emphasized the following categories when analyzing the barriers to CAR-T use: product-related, clinical outcomes, cost-benefit, reimbursement challenges (26, 29). The study’s findings will be discussed and put into context according to these three subdomains. It should, however, be noted that the domains are interrelated and may overlap.

### 4.1 Patient-related preconditions

#### 4.1.1 Information for patients and physicians (Cluster 1), supportive network (Cluster 2), eligibility of patients (Cluster 3)

To receive CAR-T cell therapy, patients need to be eligible (Cluster 3 *Eligibility of patients*) (26). As an example, according to the German guideline for treatment of DLBCL, patients are recently categorized into “CAR-T eligible” versus “CAR-T ineligible”. Participants emphasized that a “rapid clarification”, also by referring physicians, is important, as they diagnose and assess eligibility. Kansagra et al. examined barriers to CAR-T referral and pointed out the importance of a timely referral to initiate treatment at an earlier stage. Moreover, they mention the challenge of selecting eligible patients, which is guided by eligibility criteria assessing a variety of patient-related factors (28). As mentioned by participants in Cluster 3 *Eligibility of patients*, using risk scores can facilitate this process and promote the application of guideline criteria.

From a patient’s point of view, receiving multi-facetted support contributes to the patient-friendliness of a process (35), also reflected in the cluster map as Cluster 2 *Supportive network* and Cluster 10 *Patient-oriented processes* were close together. Availability of care-givers to (emotionally) support the patient is crucial for therapy adherence (28).

This is also linked to informing patients (Cluster 1 *Information for patients and physicians*) about all relevant aspects of the treatment, starting with a *shared decision making* in the potential CAR-T process. As Ogden et al. pointed out, shared responsibility in a treatment process is crucial for patient-centered care. In practice, decision aids facilitate shared decision-making and aim to improve communication, increase patient knowledge and reduce passive decision-making (35, 36). Health literacy, as mentioned in Cluster 2 *Supportive network*, is to be anticipated when informing patients to optimally support them. A patient passport may facilitate keeping an overview from the patients and the physicians’ view. In addition, introducing a patient passport was rated as the most feasible statement indicating low efforts required to put them into practice.

The domain of patient-related preconditions showed that identifying eligible patients in time is crucial to increase overall access. Once identified, patients need to be sufficiently informed, supported and incorporated in the CAR-T process promoting the best possible outcomes.

### 4.2 System-related preconditions

#### 4.2.1 Evidence, communication and transparency (Cluster 4) paperwork (Cluster 5), interface with pharmaceutical manufacturer (Cluster 6), reimbursement (Cluster 7)

It has been observed that statements in the category (Go-Zone quadrant) “above-average important but below-average feasible” often were system-related factors that cannot be directly amended by providers themselves.

The *interface with pharmaceutical manufacturers* (Cluster 6) is crucial as the pharmaceutical manufacturers play a more active role in CAR-T cell therapy compared to, e.g. over-the-counter drugs. Since CAR-T cell products are manufactured for individual patients, a well-working, user friendly system is crucial. Collaboration and cooperation with manufacturers are more important compared to ready to apply drugs. Subgroup analysis showed that participants in the above-average cases per year group (>30 cases per year) perceived this cluster as less feasible. This could be due to the fact that an increasing number of cases requires greater coordination with different CAR-T product manufacturers who have heterogeneous processes in place.

As mentioned before, assessing the eligibility of patients is not always unambiguous. In such indistinct cases, individual applications are sent to the MD (Medizinischer Dienst; engl. Medical Service) for case-by-case assessment to avoid recourses and a lack of cost-coverage. The MD is the appraisal body of the statutory health insurances in Germany and examines individual cases regarding medical indication (in- or off-label) and cost containment. Off-label use of CAR-T products may result in MD recourses, increasing the bureaucratic burden of CAR-T therapy further (Cluster 5 *Paperwork*) while decreasing the certainty of *reimbursement* (Cluster 7). Clear guidelines were mentioned in Cluster 4 *Evidence, communication and transparency* as crucial to reduce uncertainties and related paperwork.

As a prominent finding of our study, *reimbursement* (Cluster 7) was identified as the least feasible but third most important cluster. Sufficient and rapid realization of fundamental reimbursement as a precondition for the diffusion of an innovation has been discussed across various indications and therapies (37, 38). With an average drug price for CAR-T products across markets of approximately $350,000 (9) [Germany: €239,000 - €420,000 depending on the product (39)], an assured reimbursement becomes even more crucial for clinics to avoid financial risks. Therefore, clinics may decide to clarify cost coverage with sickness funds in advance for in-label use of CAR-T as well. Whereas some clinics initiate the order process once the sickness fund confirms the in-label indication, others will only order after the sickness fund declared full cost-coverage. The burden of financial risks for providers and the lack of sufficient reimbursement has been described across countries and continents [e.g., (23, 26–28)].

In contrast, novel and mostly high-priced therapies burden limited health budgets. The estimated impact of introducing CAR-T for German SHI budget over 6 years amounts to at least €448 million (40). Approaches to this issue are the topic of current (health economic) debates. One option could be demonstrating the cost-effectiveness ratio and budget impact by weighing costs and consequences. Another concept discussed is outcomes-based reimbursement (OBR) (9). Collecting (real-world) evidence as mentioned in Cluster 4 *Evidence, communication and transparency*, is the foundation for the development of such value-based pricing schemes. As described by Jørgensen et al., European countries are moving towards OBR models (41). It is clear, that reimbursement of CAR-Ts as an ATMP cannot be tackled without proper evidence for its benefits in relation to costs. Kurte et al. and Jakobs et al., for instance, have assessed the cost-effectiveness of DLBCL treatment alternatives, including CAR-T (38, 42).

In general, there is access to CAR-T therapy in Germany and Europe. However, mere approval of an intervention on a national level does not equal sufficient reimbursement and frictionless administrative processes at clinic level (9).

### 4.3 Provider-related preconditions

#### 4.3.1 Quality management (Cluster 8), infrastructure of CAR-T clinics (Cluster 9), patient-oriented processes (Cluster 10)

When it comes to provider-related factors, the mentioned aspects can apply to outpatient and inpatient sector. As assessed, *good cooperation and communication between the various institutions and professions* (Statement 58) and *good integration of referring physicians* (Statement 43) is important for a frictionless CAR-T process.

*Quality management* (Cluster 8) often includes aspects of standardization, digitalization, and collaboration but also training and timeliness of the process. Reducing hurdles that delay the process is of high importance. For instance, a short vein-to-vein time (time from apheresis to infusion) as mentioned by participants, is crucial as patients may become ineligible in the meantime, which is in line with Gajra et al. (26).

*Infrastructure of CAR-T clinics* (Cluster 9) is related to capacities (personnel, beds, ICU capacity, apheresis slots). Another aspect mentioned was the existence of CAR-T coordinators who are coordinating the entire workflow of the CAR-T process as well as supporting patients and physicians along the therapy steps. Examples from other oncologic indications have demonstrated the benefits of introducing care coordinators, such as improved quality and timeliness of care, increased patients’ satisfaction, and potential cost savings (43, 44). The introduction of CAR-T coordinators in some German CAR-T clinics is one example showing that the process has already been improved over time.

In Germany, clinics need to undergo a certification process in order to be allowed to perform CAR-T therapy. This is an aspect combining quality assurance and sufficient infrastructure as a prerequisite for high quality (25).

Cluster 10 *Patient-oriented processes* – rated as the most important cluster – is an overlap between provider and patient-related aspects which was underlined by their proximity on the cluster map. As this cluster was perceived to be the most important, promoting patient-oriented processes should be prioritized when creating and amending the CAR-T process. This is supported by Kwame et al. mentioning that for patient-orientation, effective communication and sufficient (nursing) staff capacities are required to realize the respective processes which, in turn, increase care outcomes (45). The concept mapping analysis by Ogden et al. identified requirements for patient-centered care, underlining and confirming the importance of this topic. Their analysis resulted in 13 clusters within the following three domains: a) Humanity and partnership, b) Career spanning education and training, and c) Health system, policy and management. The latter includes, e.g., resources for coordination of care as well as commitment to supportive structures and processes (35).

In recent years, outpatient administration of CAR-T therapy (if appropriate for a patient’s clinical risk profile) has evolved and expanded. As Hansen et al. reported in their systematic review, clinical and economic outcomes in the inpatient and outpatient CAR-T setting are comparable, thus paving the way for an outpatient, patient-oriented CAR-T process while at the same time reducing resource consumption (46).

The combined achievement of patient-orientation, sufficient capacities, and assured quality (which condition each other) may lead to an optimization of the CAR-T process. In return, involved personnel and patients will benefit from smooth processes and high treatment quality.

### 4.4 Next steps

In the GCM methodology described by Kane and Trochim, the analysis is followed by the utilization of the findings including the derivation of measures (31) – here taking into account importance and feasibility. In this case, the cluster map is the foundation for consecutive strategies and practical measures to promote the existence of identified preconditions. After finishing the analysis, results have been shown to and discussed with participants. They reported to agree with the concept map. Future steps are currently being discussed, including formation of working groups for the implementation of measures and, if necessary, initiating political discussions.

### 4.5 Limitations

Qualitative aspects of this mixed-methods approach have inherent limitations regarding subjectivity. When, e.g., selecting the final number of clusters, there is not only one correct solution. Authors have, however, discussed issues and collaboratively found consensus.

Another aspect of the qualitative data collection in this GCM analysis is the lack of in-depth interviews and thus the limited opportunity for participants to explain the reasoning for the statements mentioned. Conducting expert interviews could be the subject of future research.

When interpreting the importance and feasibility of statements and clusters, it should be noted that average ratings within the two dimensions are relatively close to each other. For instance, average cluster importance ratings are all above 4.0 (on a 1-5 rating scale).

When creating the cluster map as a framework of common ideas and understandings, some isolated statements were included in a cluster that may not fit optimally and increase heterogeneity. In GCM, this is indicated by high bridging values. In general, it is possible to move a statement to another cluster if reasonable. However, this can potentially lead to undesired overlapping of clusters.

It should be noted that the cluster map represents the perceived similarity of statements, however, existing dependencies are not captured by the analysis.

The number of participants and CAR-T clinics were limited. This is influencing subgroup analyses as splitting the total number of 20 participants does quickly lead to one-digit samples. However, we tried to reflect a representative perspective by involving participants from different types of clinics and practices. As the total number of CAR-T experts in Germany is limited itself, the sample size is reasonable. The analysis focused on providers and process-involved stakeholders but did not survey patients. Assessing the process from a patient-perspective would have contributed to this research. Even though stakeholders from a pharmaceutical manufacturer participated, (hospital) pharmacists did not take part. Marzal-Alfaro et al. have already described the pharmacists role along the CAR-T process, resulting in a respective guide for CAR-T therapy management for pharmacists (47).

Finally, as our analysis included German stakeholders only, the external validity is limited. The findings represent insights from a high-income country with a rather well-established CAR-T process that has already undergone improvements since its introduction. The transferability to low- and middle-income countries is only possible to a limited extent. However, with an increasing number of CAR-T cell therapies across geographies and indications, other countries can learn from initial difficulties.

### 4.6 Future research

Future research should focus on the development of concrete strategies to implement/guarantee the presence of the identified preconditions. One potential next step is to set up working groups discussing the results and develop strategies and activities/measures towards a frictionless process. In accordance with the *Plan-Do-Study-Act* cycle for quality improvement, the planning phase should be followed by the implementation of measures, their evaluation, and adaptions where necessary (48).

Procedural differences between large (academic) centers and small practices, and between countries should be assessed in future research activities. Procedural differences may also occur depending on the specific CAR-T product. By conducting comparable studies with a larger sample size (e.g. multi-national stakeholders), further and more detailed subgroup analyses could be conducted. Another aspect to study in the future should be process hurdles from a patient perspective, identifying potential differences based on, e.g., socio-economic status.As dependencies between statements and clusters are not covered by this analysis, future studies should investigate how the change of one aspect influences others.

From an economic viewpoint, the results offer a basis for further analyses quantifying the economic burden of current process inefficiencies (e.g., extra costs caused by administrative efforts or the certification process), especially as *reimbursement* demonstrated discrepancies between importance and feasibility. This will further contribute to a deeper understanding of the importance regarding process optimizations.

### 4.7 Conclusion

This group concept mapping analysis empirically assessed preconditions for a frictionless CAR-T process. The results support current debates and contribute to the improvement of the process potentially leading to higher care quality and reduction of efficiency losses. It enables stakeholders and (future) practitioners to understand the process complexity and necessities and, in turn, to develop comprehensive strategies to tackle process friction.

In an international context, our analysis gives valuable insights for other countries performing or introducing CAR-T therapy. In Germany, CAR-T therapy is an established option in the more than 40 treatment centers and internal structures have been built and applied over the last six years. Learning from experiences of CAR-T experts about what barriers still exist and what is needed for a (more) frictionless process can guide the establishment and management of the process to increase effectiveness, reduce hurdles and, most importantly, improve patient outcomes.

## Data Availability

The original contributions presented in the study are included in the article/**Supplementary Material**. Further inquiries can be directed to the corresponding author.

## Footnotes

1 NUB (“Neue Untersuchungs- und Behandlungsmethoden”) = new examination and treatment methods; NUB fees are a reimbursement in addition to DRG flat fees. The aim is to remunerate novel interventions that are not yet considered in the DRG system. In Germany, NUB fees are negotiated individually between clinic and statutory health insurance.
