# Supplementary material for: How to optimize the CAR-T Cell therapy process? A group concept mapping analysis of preconditions for a frictionless process from a German multistakeholder perspective
Source: Front Oncol. 2024 Sep 23;14:1466803. doi: 10.3389/fonc.2024.1466803 (PMC11456517; doi:10.3389/fonc.2024.1466803)
Supplement: Supplementary file 1 [file DataSheet1.docx]

Supplementary Material

[Supplementary Table 1: Final statement list (incl. translation) 2](#_Toc174696336)

[Supplementary Table 2: Cluster map options 5](#_Toc174696337)

[Supplementary Table 3: Bridging values per statement and cluster 8](#_Toc174696338)

[Supplementary Table 4: Statements per quadrant 11](#_Toc174696339)

[Supplementary Figure 1: Pattern Match - Feasibility rating (above versus below average case numbers per year) 15](#_Toc174696353)

[Supplementary Figure 2: Pattern Match - Importance rating (above versus below average case numbers per year) 16](#_Toc174696354)

Supplementary Table 1: Final statement list (incl. translation)

| Statement # | German | English |
| --- | --- | --- |
| 1 | Zielgruppen- und produktspezifische Informationen (für u.a. Zuweiser*innen, Patient*innen, Behandelnde, Pflege) | Target group and product-specific information (for referring physicians, patients, practitioners, nursing professionals, etc.) |
| 2 | Nutzung von digitalen Lösungen wie Telemedizin, elektronische Patientenakte (ePA) und Apps | Use of digital solutions such as telemedicine, electronic patient records and apps |
| 3 | Juristisch abgesicherte Cancellation Policy | Juristically secured cancellation policy |
| 4 | Ausreichend Fachpersonal / Personalkapazitäten | Sufficient qualified personnel / personnel capacities |
| 5 | Klar definierte Ansprechpersonen / Kontaktmöglichkeiten für alle involvierten Personen (inkl. Patient*innen) | Clearly defined contact persons / contact options for all persons concerned (including patients) |
| 6 | Antizipierung von zukünftigen Entwicklungen (z.B. Wachstum) und Investitionen in diese | Anticipation of and investment in future developments (e.g. growth) |
| 7 | Leitlinien für spezifische Krebserkrankungen (z.B. DLBCL, CLL) | Guidelines regarding specific cancers (e.g. DLBCL, CLL) |
| 8 | Beutel mit ausreichend Volumen | Bags with sufficient volume |
| 9 | MD Management (Medizinischer Dienst) | Managing issues related to the Health Insurance Medical Service (Medizinischer Dienst; MD) |
| 10 | Patient*innen in gutem Allgemeinzustand ("fit") | Patients in overall good state of health ("fit") |
| 11 | Einfach anwendbare Risiko Scores, um eine differenzierte Vorgehensweise zu ermöglichen im ambulanten und stationären Setting | Easily applicable risk scores to enable a differentiated approach in both outpatient and inpatient settings |
| 12 | Strukturierte, sektorenübergreifende Nachsorgeprogramme | Structured, cross-sector aftercare programs |
| 13 | Zeitnahe Diagnostik | Timely diagnostics |
| 14 | Cancer Survivorship Maßnahmen | Cancer survivorship measures |
| 15 | Soziale Unterstützungsangebote | Social support services |
| 16 | Vorhalten von Kapazitäten für (Langzeit-)Nebenwirkungsmanagement | Provision of capacities for (long-term) side effect management |
| 17 | Unterstützung von Patient*innen im Vorfeld der Therapie | Support for patients in the pre-treatment phase |
| 18 | Outcome-Datenerfassung im Therapieverlauf und der Nachsorge | Outcome data collection during the course of therapy and aftercare |
| 19 | Rechtssicherheit zur Vermeidung von Regressen (Medizinischer Dienst) und damit verbundene Erstattungssicherheit | Legal certainty to avoid recourses by the Health Insurance Medical Service (Medizinischer Dienst; MD) and the associated certainty of reimbursement |
| 20 | Wenig Personalwechsel beim "Nachsorgepersonal" | Few changes in "aftercare personnel" |
| 21 | Einen Patient*innenpass | A patient passport |
| 22 | Regelmäßige Informationen über Forschungsvorhaben und Studienteilnahmen für die Patient*innenrekrutierung | Regular information on research projects and study participation for patient recruitment |
| 23 | Mehr Transparenz bei der Entwicklung der Erstattungsbeträge nach dem AMNOG-Verfahren | More transparency in the development of reimbursement amounts following the AMNOG procedure |
| 24 | Gesundheitskompetenz der Patient*innen | Patients´ health literacy |
| 25 | Shared decision making | Shared decision making |
| 26 | Bettenverfügbarkeit Normalstation | Bed availability on normal ward |
| 27 | Bettenverfügbarkeit Intensivstation | Bed availability on intensive care unit |
| 28 | Unterstützung aus dem privaten/familiären Umfeld der Patient*innen | Support from the patient´s private/family environment |
| 29 | Standardisierte Maßnahmen und Materialien zur Dokumentation | Standardized measures and materials for documentation |
| 30 | Gutes Projektmanagement und Standard Operating Procedures (SOP) | Good project management and standard operating procedures (SOP) |
| 31 | Berücksichtigung von Unterschieden in real-world Populationen versus Studienpopulationen | Consideration of differences in real-world populations vs. study populations |
| 32 | Sektorenspezifische, einheitliche Kodierregeln | Sector-specific, standardized coding rules |
| 33 | Qualitätssicherung und -management | Quality assurance and management |
| 34 | Spezifische DRG(s) für CAR-T-Zelltherapie | Specific DRG(s) for CAR-T cell therapy |
| 35 | Ausreichende und deutschlandweit einheitliche Refinanzierung (zusätzliches NUB-Entgelt) | Sufficient and uniform nationwide refinancing (additional NUB fee) |
| 36 | Pauschale für Etablierungs- und Vorhaltungskosten | Lump sum for establishment and maintenance costs |
| 37 | Sektorenübergreifende adäquate Finanzierung | Adequate financing across sectors |
| 38 | Erfahrungsaustausch | Exchange of experiences |
| 39 | Gute Kooperation und Kommunikation innerhalb der Institutionen | Good cooperation and communication within the institutions |
| 40 | Gute Einbindung des Pflegepersonals | Good involvement of nursing personnel |
| 41 | Koordiniertes Entlassmanagement | Coordinated discharge management |
| 42 | Gute Anbindung an das CAR-T-Zentrum | Good connection/ access to the CAR-T center |
| 43 | Gute Einbindung der Zuweiser*innen | Good integration of referring physicians |
| 44 | CAR-T-Zell Koordinator*innen/ Lots*innen | CAR-T cell coordinators/guides |
| 45 | Überbrückung der Zeit durch angemessene Bridging-Therapien | Bridging the time through appropriate bridging therapies |
| 46 | Möglichst kurze Zeiten zwischen Fertigstellung des Produkts und Produktabholung | Minimum possible time between completion of the product and product delivery |
| 47 | Vermeidung von Infekten, die den Prozess verzögern würden | Avoidance of infections that would delay the process |
| 48 | Einbeziehen der Angehörigen in Erläuterung medizinischer Sachverhalte | Involvement of relatives in the explanation of medical issues |
| 49 | Kenntnisse über und fokussierte Hinweise auf das im Verlauf mögliche Nebenwirkungsspektrum | Knowledge of and focused information on the possible spectrum of side effects during the course of the disease |
| 50 | Ambulante spezialfachärztliche Versorgung (ASV) von Hämatoonkolog*innen | Outpatient specialist care (German: ambulante spezialfachärztliche Versorgung - ASV) for hemato-oncologists (§116b Social Codebook V) |
| 51 | Selektivvertragliche Lösungen | Selective contractual agreements (§ 140a Social Codebook V) |
| 52 | Checklisten für Indikationsstellung und Voruntersuchungen | Checklists for indications and preliminary medical examination |
| 53 | Spezialisierte CAR-T-Zentren | Specialized CAR-T centers |
| 54 | Kostenübernahmesicherheit in allen Sektoren | Security of cost coverage in all sectors |
| 55 | Gemeinsame Tumorboards | Collaborative tumor boards |
| 56 | Zügige Abklärung der Eignung der Patient*innen für die CAR-T-Therapie | Rapid clarification of the patient's suitability for CAR-T therapy |
| 57 | Inhouse Apheraseeinheiten / Apheresenetzwerke | In-house apheresis units / apheresis networks |
| 58 | Gute Kooperation und Kommunikation zwischen den einzelnen Institutionen und Professionen | Good cooperation and communication between the various institutions and professions |
| 59 | Reduktion des bürokratischen Aufwands | Reduction of the bureaucratic burden |
| 60 | Ausreichende Apheresekapazitäten | Sufficient apheresis capacities |
| 61 | Industrieübergreifende Standards | Cross-industry standards |
| 62 | Informationen über Zytostatika, die die Apherese beeinflussen können | Information on cytostatic drugs that can affect apheresis |
| 63 | Individuelle Aufklärung der Patient*innen | Individual information for patients |
| 64 | Nutzerfreundliches Bestellportal | User-friendly ordering portal |
| 65 | Aufnahme der Apherese in die G-BA-Richtlinie | Inclusion of apheresis in the joint federal committee (German: Gemeinsamer Bundesausschuss; G-BA) guideline |
| 66 | Herstellungskapazitäten in Europa | Manufacturing capacities in Europe |
| 67 | Transparenz bei der Out-of-Specification (OOS) | Transparency concerning out-of-specification (OOS) |
| 68 | Inhouse Kryokonservierung | In-house cryopreservation |
| 69 | Transparentes, digitales Prozesstracking | Transparent, digital process tracking |
| 70 | Kurze Vein-to-Vein-Zeit | Short vein-to-vein time |
| 71 | Digitalisierung des Prozesses | Digitization of the process |
| 72 | Weiterbildungen für am Prozess beteiligtes Personal | Further training for personnel involved in the process |
| 73 | Ambulante Behandlung, sofern möglich (z.B. auch ambulante Lymphdepletion) | Outpatient treatment, if possible (e.g. also outpatient lymphatic depletion) |
| 74 | Vertraglich geregelte Zusammenarbeit | Contractually regulated collaboration |
| 75 | Effiziente Kommunikation bzgl. Änderungen der Timeline durch Bridging-Therapie | Efficient communication regarding changes to the timeline through bridging therapy |
| 76 | Qualitätsmanagement bzgl. des Produkts vonseiten der Apotheke | Quality management of the product by the pharmacy |
| 77 | Interdisziplinäre Teams | Interdisciplinary teams |
| 78 | Lieferung ins Zelllabor durch Apotheke | Delivery to the cell laboratory by pharmacy |
| 79 | Vorhaltung in der Apotheke | Stocking in the pharmacy |
| 80 | Eine klare Definition der Zweitlinientherapie | A clear definition for second-line therapy |

Supplementary Table 2: Cluster map options

**Table 2a: Available cluster map options and merging information**

| **Cluster count ↓** |  |  |  |  |  |  |  |  |  |  |  |  |  |  |  |
| --- | --- | --- | --- | --- | --- | --- | --- | --- | --- | --- | --- | --- | --- | --- | --- |
| 15 | C1 | C2 | C3 | C4 | C5 | C6 | C7 | C8 | C9 | C10 | C11 | C12 | C13 | C14 | C15 |
| 14 | C1 | C2 | C3 | merge: C4 | | C5 | C6 | C7 | C8 | C9 | C10 | C11 | C12 | C13 | C14 |
| 13 | C1 | C2 | C3 | C4 | | C5 | C6 | C7 | C8 | merge: C9 | | C10 | C11 | C12 | C13 |
| 12 | C1 | C2 | C3 | C4 | | C5 | C6 | C7 | C8 | C9 | | C10 | C11 | merge: C12 | |
| 11 | C1 | C2 | C3 | C4 | | C5 | C6 | C7 | C8 | C9 | | merge: C10 | | C11 | |
| 10 | C1 | C2 | C3 | C4 | | C5 | C6 | C7 | merge: C8 | | | C9 | | C10 | |
| 9 | C1 | C2 | C3 | C4 | | merge: C5 | | C6 | C7 | | | C8 | | C9 | |
| 8 | merge: C1 | | C2 | C3 | | C4 | | C5 | C6 | | | C7 | | C8 | |
| 7 | merge: C1 | | | C2 | | C3 | | C4 | C5 | | | C6 | | C7 | |
| 6 | C1 | | | C2 | | merge: C3 | | | C4 | | | C5 | | C6 | |
| 5 | C1 | | | C2 | | C3 | | | merge: C4 | | | | | C5 | |
| 4 | merge: C1 | | | | | C2 | | | C3 | | | | | C4 | |

Table 2a displays all available cluster options which were reviewed as possible cluster maps. Cluster map options ranged from 15 to 4 clusters. Each row reflects one cluster map option with the respective count of clusters. Going from one row to the consecutive row below, the table shows which clusters from the previous solution were merged. (For example: The 14-cluster-solution includes 14 clusters as Cluster 4 (C4) and Cluster 5 (C5) from the 15-cluster solution were merged.) Finally, the 10-cluster-solution was chosen. By combining Table 2a and 2b, each cluster map option can be deducted.

**Table 2b: Statement distribution in 15-cluster-map solution**

| Cluster | Statement ID | Statement |
| --- | --- | --- |
| C1 | 1 | Target group and product-specific information (for referring physicians, patients, practitioners, nursing professionals, etc.) |
| C1 | 25 | Shared decision making |
| C1 | 31 | Consideration of differences in real-world populations vs. study populations |
| C1 | 49 | Knowledge of and focused information on the possible spectrum of side effects during the course of the disease |
| C2 | 14 | Cancer survivorship measures |
| C2 | 15 | Social support services |
| C2 | 17 | Support for patients in the pre-treatment phase |
| C2 | 21 | A patient passport |
| C2 | 24 | Patients´ health literacy |
| C2 | 28 | Support from the patient´s private/family environment |
| C2 | 48 | Involvement of relatives in the explanation of medical issues |
| C3 | 10 | Patients in overall good state of health ("fit") |
| C3 | 11 | Easily applicable risk scores to enable a differentiated approach in both outpatient and inpatient settings |
| C3 | 56 | Rapid clarification of the patient's suitability for CAR-T therapy |
| C3 | 62 | Information on cytostatic drugs that can affect apheresis |
| C3 | 63 | Individual information for patients |
| C4 | 7 | Guidelines regarding specific cancers (e.g. DLBCL, CLL) |
| C4 | 67 | Transparency concerning out-of-specification (OOS) |
| C4 | 74 | Contractually regulated collaboration |
| C5 | 8 | Bags with sufficient volume |
| C5 | 18 | Outcome data collection during the course of therapy and aftercare |
| C5 | 22 | Regular information on research projects and study participation for patient recruitment |
| C5 | 80 | A clear definition for second-line therapy |
| C6 | 3 | Juristically secured cancellation policy |
| C6 | 9 | Managing issues related to the Health Insurance Medical Service (Medizinischer Dienst; MD) |
| C6 | 59 | Reduction of the bureaucratic burden |
| C6 | 65 | Inclusion of apheresis in the joint federal committee (German: Gemeinsamer Bundesausschuss; G-BA) guideline |
| C7 | 50 | Outpatient specialist care (German: ambulante spezialfachärztliche Versorgung - ASV) for hemato-oncologists (§116b Social Codebook V) |
| C7 | 61 | Cross-industry standards |
| C7 | 64 | User-friendly ordering portal |
| C7 | 66 | Manufacturing capacities in Europe |
| C8 | 19 | Legal certainty to avoid recourses by the Health Insurance Medical Service (Medizinischer Dienst; MD) and the associated certainty of reimbursement |
| C8 | 23 | More transparency in the development of reimbursement amounts following the AMNOG procedure |
| C8 | 32 | Sector-specific, standardized coding rules |
| C8 | 34 | Specific DRG(s) for CAR-T cell therapy |
| C8 | 35 | Sufficient and uniform nationwide refinancing (additional NUB fee) |
| C8 | 36 | Lump sum for establishment and maintenance costs |
| C8 | 37 | Adequate financing across sectors |
| C8 | 51 | Selective contractual agreements (§ 140a Social Codebook V) |
| C8 | 54 | Security of cost coverage in all sectors |
| C9 | 2 | Use of digital solutions such as telemedicine, electronic patient records and apps |
| C9 | 38 | Exchange of experiences |
| C9 | 71 | Digitization of the process |
| C10 | 6 | Anticipation of and investment in future developments (e.g. growth) |
| C10 | 29 | Standardized measures and materials for documentation |
| C10 | 46 | Minimum possible time between completion of the product and product delivery |
| C10 | 69 | Transparent, digital process tracking |
| C11 | 30 | Good project management and standard operating procedures (SOP) |
| C11 | 33 | Quality assurance and management |
| C11 | 55 | Collaborative tumor boards |
| C11 | 70 | Short vein-to-vein time |
| C11 | 72 | Further training for personnel involved in the process |
| C11 | 76 | Quality management of the product by the pharmacy |
| C12 | 4 | Sufficient qualified personnel / personnel capacities |
| C12 | 16 | Provision of capacities for (long-term) side effect management |
| C12 | 20 | Few changes in "aftercare personnel" |
| C12 | 26 | Bed availability on normal ward |
| C12 | 39 | Good cooperation and communication within the institutions |
| C12 | 40 | Good involvement of nursing personnel |
| C12 | 41 | Coordinated discharge management |
| C12 | 44 | CAR-T cell coordinators/guides |
| C12 | 53 | Specialized CAR-T centers |
| C12 | 77 | Interdisciplinary teams |
| C13 | 27 | Bed availability on intensive care unit |
| C13 | 57 | In-house apheresis units / apheresis networks |
| C13 | 60 | Sufficient apheresis capacities |
| C13 | 68 | In-house cryopreservation |
| C13 | 78 | Delivery to the cell laboratory by pharmacy |
| C13 | 79 | Stocking in the pharmacy |
| C14 | 5 | Clearly defined contact persons / contact options for all persons concerned (including patients) |
| C14 | 13 | Timely diagnostics |
| C14 | 43 | Good integration of referring physicians |
| C14 | 58 | Good cooperation and communication between the various institutions and professions |
| C14 | 73 | Outpatient treatment, if possible (e.g. also outpatient lymphatic depletion) |
| C15 | 12 | Structured, cross-sector aftercare programs |
| C15 | 42 | Good connection/ access to the CAR-T center |
| C15 | 45 | Bridging the time through appropriate bridging therapies |
| C15 | 47 | Avoidance of infections that would delay the process |
| C15 | 52 | Checklists for indications and preliminary medical examination |
| C15 | 75 | Efficient communication regarding changes to the timeline through bridging therapy |

Supplementary Table 3: Bridging values per statement and cluster

| **Statement ID** | **Statement** | **Bridging value** |
| --- | --- | --- |
| **Cluster 1** | **Information for patients and physicians** | **Avg 0.7** |
| 1 | Target group and product-specific information (for referring physicians, patients, practitioners, nursing professionals, etc.) | 0.79 |
| 25 | Shared decision making | 0.61 |
| 31 | Consideration of differences in real-world populations vs. study populations | 0.67 |
| 49 | Knowledge of and focused information on the possible spectrum of side effects during the course of the disease | 0.73 |
| **Cluster 2** | **Supportive network** | **Avg 0.44** |
| 14 | Cancer survivorship measures | 0.62 |
| 15 | Social support services | 0.47 |
| 17 | Support for patients in the pre-treatment phase | 0.4 |
| 21 | A patient passport | 0.45 |
| 24 | Patients´ health literacy | 0.39 |
| 28 | Support from the patient´s private/family environment | 0.37 |
| 48 | Involvement of relatives in the explanation of medical issues | 0.39 |
| **Cluster 3** | **Eligibility of patients** | **Avg 0.49** |
| 10 | Patients in overall good state of health ("fit") | 0.47 |
| 11 | Easily applicable risk scores to enable a differentiated approach in both outpatient and inpatient settings | 0.49 |
| 56 | Rapid clarification of the patient's suitability for CAR-T therapy | 0.49 |
| 62 | Information on cytostatic drugs that can affect apheresis | 0.51 |
| 63 | Individual information for patients | 0.47 |
| **Cluster 4** | **Evidence, communication and transparency** | **Avg 0.78** |
| 7 | Guidelines regarding specific cancers (e.g. DLBCL, CLL) | 0.83 |
| 8 | Bags with sufficient volume | 0.95 |
| 18 | Outcome data collection during the course of therapy and aftercare | 0.82 |
| 22 | Regular information on research projects and study participation for patient recruitment | 0.64 |
| 67 | Transparency concerning out-of-specification (OOS) | 0.67 |
| 74 | Contractually regulated collaboration | 1 |
| 80 | A clear definition for second-line therapy | 0.57 |
| **Cluster 5** | **Paperwork** | **Avg 0.4** |
| 3 | Juristically secured cancellation policy | 0.4 |
| 9 | Managing issues related to the Health Insurance Medical Service (Medizinischer Dienst; MD) | 0.36 |
| 59 | Reduction of the bureaucratic burden | 0.49 |
| 65 | Inclusion of apheresis in the joint federal committee (German: Gemeinsamer Bundesausschuss; G-BA) guideline | 0.35 |
| **Cluster 6** | **Interface with pharmaceutical manufacturer** | **Avg 0.62** |
| 50 | Outpatient specialist care (German: ambulante spezialfachärztliche Versorgung - ASV) for hemato-oncologists (§116b Social Codebook V) | 0.67 |
| 61 | Cross-industry standards | 0.66 |
| 64 | User-friendly ordering portal | 0.6 |
| 66 | Manufacturing capacities in Europe | 0.57 |
| **Cluster 7** | **Reimbursement** | **Avg 0.08** |
| 19 | Legal certainty to avoid recourses by the Health Insurance Medical Service (Medizinischer Dienst; MD) and the associated certainty of reimbursement | 0.07 |
| 23 | More transparency in the development of reimbursement amounts following the AMNOG procedure | 0.03 |
| 32 | Sector-specific, standardized coding rules | 0.06 |
| 34 | Specific DRG(s) for CAR-T cell therapy | 0 |
| 35 | Sufficient and uniform nationwide refinancing (additional NUB fee) | 0.08 |
| 36 | Lump sum for establishment and maintenance costs | 0.02 |
| 37 | Adequate financing across sectors | 0.19 |
| 51 | Selective contractual agreements (§ 140a Social Codebook V) | 0.17 |
| 54 | Security of cost coverage in all sectors | 0.08 |
| **Cluster 8** | **Quality Management** | **Avg 0.43** |
| 2 | Use of digital solutions such as telemedicine, electronic patient records and apps | 0.38 |
| 6 | Anticipation of and investment in future developments (e.g. growth) | 0.69 |
| 29 | Standardized measures and materials for documentation | 0.47 |
| 30 | Good project management and standard operating procedures (SOP) | 0.36 |
| 33 | Quality assurance and management | 0.39 |
| 38 | Exchange of experiences | 0.38 |
| 46 | Minimum possible time between completion of the product and product delivery | 0.62 |
| 55 | Collaborative tumor boards | 0.35 |
| 69 | Transparent, digital process tracking | 0.44 |
| 70 | Short vein-to-vein time | 0.35 |
| 71 | Digitization of the process | 0.42 |
| 72 | Further training for personnel involved in the process | 0.34 |
| 76 | Quality management of the product by the pharmacy | 0.38 |
| **Cluster 9** | **Infrastructure of CAR-T clinics** | **Avg 0.31** |
| 4 | Sufficient qualified personnel / personnel capacities | 0.19 |
| 16 | Provision of capacities for (long-term) side effect management | 0.38 |
| 20 | Few changes in "aftercare personnel" | 0.38 |
| 26 | Bed availability on normal ward | 0.24 |
| 27 | Bed availability on intensive care unit | 0.25 |
| 39 | Good cooperation and communication within the institutions | 0.24 |
| 40 | Good involvement of nursing personnel | 0.21 |
| 41 | Coordinated discharge management | 0.39 |
| 44 | CAR-T cell coordinators/guides | 0.2 |
| 53 | Specialized CAR-T centers | 0.25 |
| 57 | In-house apheresis units / apheresis networks | 0.28 |
| 60 | Sufficient apheresis capacities | 0.35 |
| 68 | In-house cryopreservation | 0.32 |
| 77 | Interdisciplinary teams | 0.21 |
| 78 | Delivery to the cell laboratory by pharmacy | 0.49 |
| 79 | Stocking in the pharmacy | 0.51 |
| **Cluster 10** | **Patient-oriented processes** | **Avg 0.41** |
| 5 | Clearly defined contact persons / contact options for all persons concerned (including patients) | 0.34 |
| 12 | Structured, cross-sector aftercare programs | 0.43 |
| 13 | Timely diagnostics | 0.42 |
| 42 | Good connection/ access to the CAR-T center | 0.42 |
| 43 | Good integration of referring physicians | 0.33 |
| 45 | Bridging the time through appropriate bridging therapies | 0.49 |
| 47 | Avoidance of infections that would delay the process | 0.39 |
| 52 | Checklists for indications and preliminary medical examination | 0.41 |
| 58 | Good cooperation and communication between the various institutions and professions | 0.32 |
| 73 | Outpatient treatment, if possible (e.g. also outpatient lymphatic depletion) | 0.47 |
| 75 | Efficient communication regarding changes to the timeline through bridging therapy | 0.5 |

Supplementary Table 4: Statements per quadrant

| Statement ID | Cluster | Statement | Importance rating (mean) | Feasibility rating (mean) | Quadrant |
| --- | --- | --- | --- | --- | --- |
| 49 | Cluster 1 | Knowledge of and focused information on the possible spectrum of side effects during the course of the disease | 4.5294 | 3.9375 | upper right |
| 5 | Cluster 10 | Clearly defined contact persons / contact options for all persons concerned (including patients) | 4.8235 | 3.6875 | upper right |
| 13 | Cluster 10 | Timely diagnostics | 4.7647 | 3.1875 | upper right |
| 42 | Cluster 10 | Good connection/ access to the CAR-T center | 4.7059 | 3.3125 | upper right |
| 43 | Cluster 10 | Good integration of referring physicians | 4.7059 | 3.3125 | upper right |
| 58 | Cluster 10 | Good cooperation and communication between the various institutions and professions | 4.5882 | 3.375 | upper right |
| 45 | Cluster 10 | Bridging the time through appropriate bridging therapies | 4.4118 | 3.6875 | upper right |
| 52 | Cluster 10 | Checklists for indications and preliminary medical examination | 4.3529 | 4 | upper right |
| 75 | Cluster 10 | Efficient communication regarding changes to the timeline through bridging therapy | 4.2353 | 3.625 | upper right |
| 28 | Cluster 2 | Support from the patient´s private/family environment | 4.3529 | 3.625 | upper right |
| 48 | Cluster 2 | Involvement of relatives in the explanation of medical issues | 4.2353 | 4.0625 | upper right |
| 56 | Cluster 3 | Rapid clarification of the patient's suitability for CAR-T therapy | 4.7059 | 3.8125 | upper right |
| 63 | Cluster 3 | Individual information for patients | 4.6471 | 4.25 | upper right |
| 62 | Cluster 3 | Information on cytostatic drugs that can affect apheresis | 4.3529 | 4.125 | upper right |
| 11 | Cluster 3 | Easily applicable risk scores to enable a differentiated approach in both outpatient and inpatient settings | 4.2353 | 3.3125 | upper right |
| 18 | Cluster 4 | Outcome data collection during the course of therapy and aftercare | 4.3529 | 3.1875 | upper right |
| 7 | Cluster 4 | Guidelines regarding specific cancers (e.g. DLBCL, CLL) | 4.2941 | 3.5 | upper right |
| 64 | Cluster 6 | User-friendly ordering portal | 4.3529 | 3.625 | upper right |
| 72 | Cluster 8 | Further training for personnel involved in the process | 4.6471 | 3.625 | upper right |
| 33 | Cluster 8 | Quality assurance and management | 4.5882 | 3.25 | upper right |
| 38 | Cluster 8 | Exchange of experiences | 4.4706 | 3.875 | upper right |
| 55 | Cluster 8 | Collaborative tumor boards | 4.3529 | 4 | upper right |
| 30 | Cluster 8 | Good project management and standard operating procedures (SOP) | 4.2941 | 3.875 | upper right |
| 40 | Cluster 9 | Good involvement of nursing personnel | 4.7059 | 3.5 | upper right |
| 44 | Cluster 9 | CAR-T cell coordinators/guides | 4.6471 | 3.375 | upper right |
| 39 | Cluster 9 | Good cooperation and communication within the institutions | 4.5882 | 3.625 | upper right |
| 53 | Cluster 9 | Specialized CAR-T centers | 4.5882 | 3.4375 | upper right |
| 77 | Cluster 9 | Interdisciplinary teams | 4.4706 | 3.5 | upper right |
| 41 | Cluster 9 | Coordinated discharge management | 4.2353 | 3.5625 | upper right |
| 1 | Cluster 1 | Target group and product-specific information (for referring physicians, patients, practitioners, nursing professionals, etc.) | 4 | 3.8125 | upper left |
| 8 | Cluster 4 | Bags with sufficient volume | 3.5 | 3.1333 | upper left |
| 14 | Cluster 2 | Cancer survivorship measures | 3.8824 | 3.1875 | upper left |
| 17 | Cluster 2 | Support for patients in the pre-treatment phase | 4.1765 | 3.4375 | upper left |
| 21 | Cluster 2 | A patient passport | 3.5294 | 4.375 | upper left |
| 22 | Cluster 4 | Regular information on research projects and study participation for patient recruitment | 3.9412 | 4 | upper left |
| 25 | Cluster 1 | Shared decision making | 4 | 3.875 | upper left |
| 29 | Cluster 8 | Standardized measures and materials for documentation | 4 | 3.5625 | upper left |
| 31 | Cluster 1 | Consideration of differences in real-world populations vs. study populations | 4.1176 | 3.4375 | upper left |
| 67 | Cluster 4 | Transparency concerning out-of-specification (OOS) | 4.1176 | 3.3125 | upper left |
| 80 | Cluster 4 | A clear definition for second-line therapy | 4.0588 | 3.4375 | upper left |
| 54 | Cluster 7 | Security of cost coverage in all sectors | 4.7647 | 2.0625 | lower right |
| 60 | Cluster 9 | Sufficient apheresis capacities | 4.7647 | 2.625 | lower right |
| 4 | Cluster 9 | Sufficient qualified personnel / personnel capacities | 4.7059 | 2.0625 | lower right |
| 35 | Cluster 7 | Sufficient and uniform nationwide refinancing (additional NUB fee) | 4.7059 | 2 | lower right |
| 37 | Cluster 7 | Adequate financing across sectors | 4.7059 | 2.1875 | lower right |
| 59 | Cluster 5 | Reduction of the bureaucratic burden | 4.6471 | 2.25 | lower right |
| 19 | Cluster 7 | Legal certainty to avoid recourses by the Health Insurance Medical Service (Medizinischer Dienst; MD) and the associated certainty of reimbursement | 4.5294 | 2.0625 | lower right |
| 46 | Cluster 8 | Minimum possible time between completion of the product and product delivery | 4.5294 | 2.75 | lower right |
| 70 | Cluster 8 | Short vein-to-vein time | 4.5294 | 2.75 | lower right |
| 12 | Cluster 10 | Structured, cross-sector aftercare programs | 4.4706 | 3.0625 | lower right |
| 47 | Cluster 10 | Avoidance of infections that would delay the process | 4.4118 | 2.8125 | lower right |
| 27 | Cluster 9 | Bed availability on intensive care unit | 4.2941 | 2.5625 | lower right |
| 34 | Cluster 7 | Specific DRG(s) for CAR-T cell therapy | 4.2353 | 2.125 | lower right |
| 2 | Cluster 8 | Use of digital solutions such as telemedicine, electronic patient records and apps | 3.6471 | 2.5 | lower left |
| 3 | Cluster 5 | Juristically secured cancellation policy | 3.9412 | 2.875 | lower left |
| 6 | Cluster 8 | Anticipation of and investment in future developments (e.g. growth) | 3.9412 | 2.875 | lower left |
| 9 | Cluster 5 | Managing issues related to the Health Insurance Medical Service (Medizinischer Dienst; MD) | 3.7647 | 2.625 | lower left |
| 10 | Cluster 3 | Patients in overall good state of health ("fit") | 4.1176 | 2.625 | lower left |
| 15 | Cluster 2 | Social support services | 3.9412 | 3.0625 | lower left |
| 16 | Cluster 9 | Provision of capacities for (long-term) side effect management | 3.8824 | 2.8125 | lower left |
| 20 | Cluster 9 | Few changes in "aftercare personnel" | 3.7647 | 2.1875 | lower left |
| 23 | Cluster 7 | More transparency in the development of reimbursement amounts following the AMNOG procedure | 4.1765 | 2.875 | lower left |
| 24 | Cluster 2 | Patients´ health literacy | 4.1176 | 2.9375 | lower left |
| 26 | Cluster 9 | Bed availability on normal ward | 4.0588 | 2.75 | lower left |

Supplementary Figure 1: Pattern Match - Feasibility rating (above versus below average case numbers per year)

| 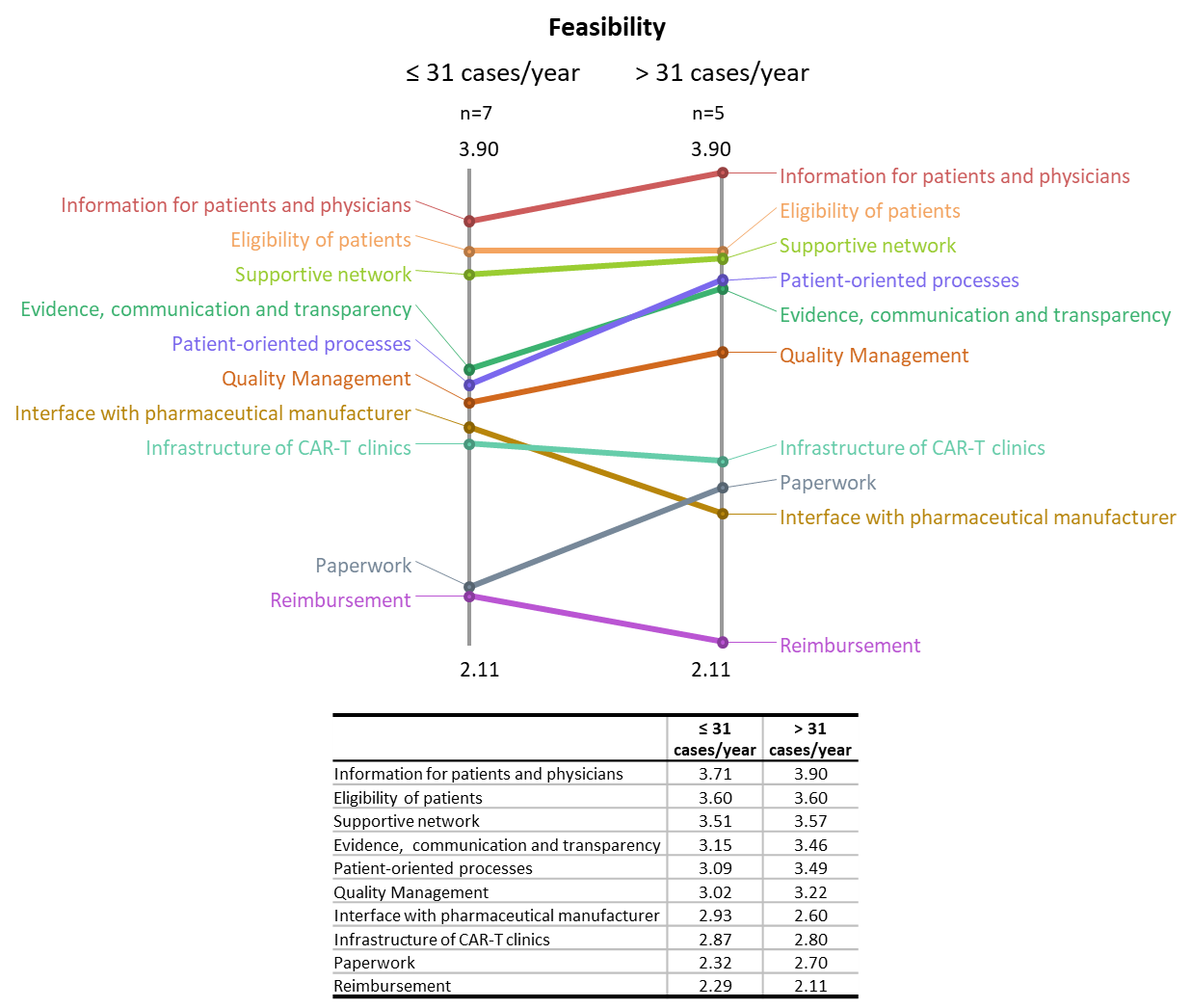 |
| --- |
| Average feasibility ratings per cluster per subgroup are contrasted. Axes values are absolute, i.e., overall minimum and maximum values (not per dimension) are reflected. Subgroups are defined based on the CAR-T cases that participants/their clinics are involved in per year (below or above the mean of 31 cases/year). 12 participants indicated a case number per year and completed the feasibility rating. Ratings were done on a 5-point Likert scale ranging from 1 to 5. |

Supplementary Figure 2: Pattern Match - Importance rating (above versus below average case numbers per year)

| 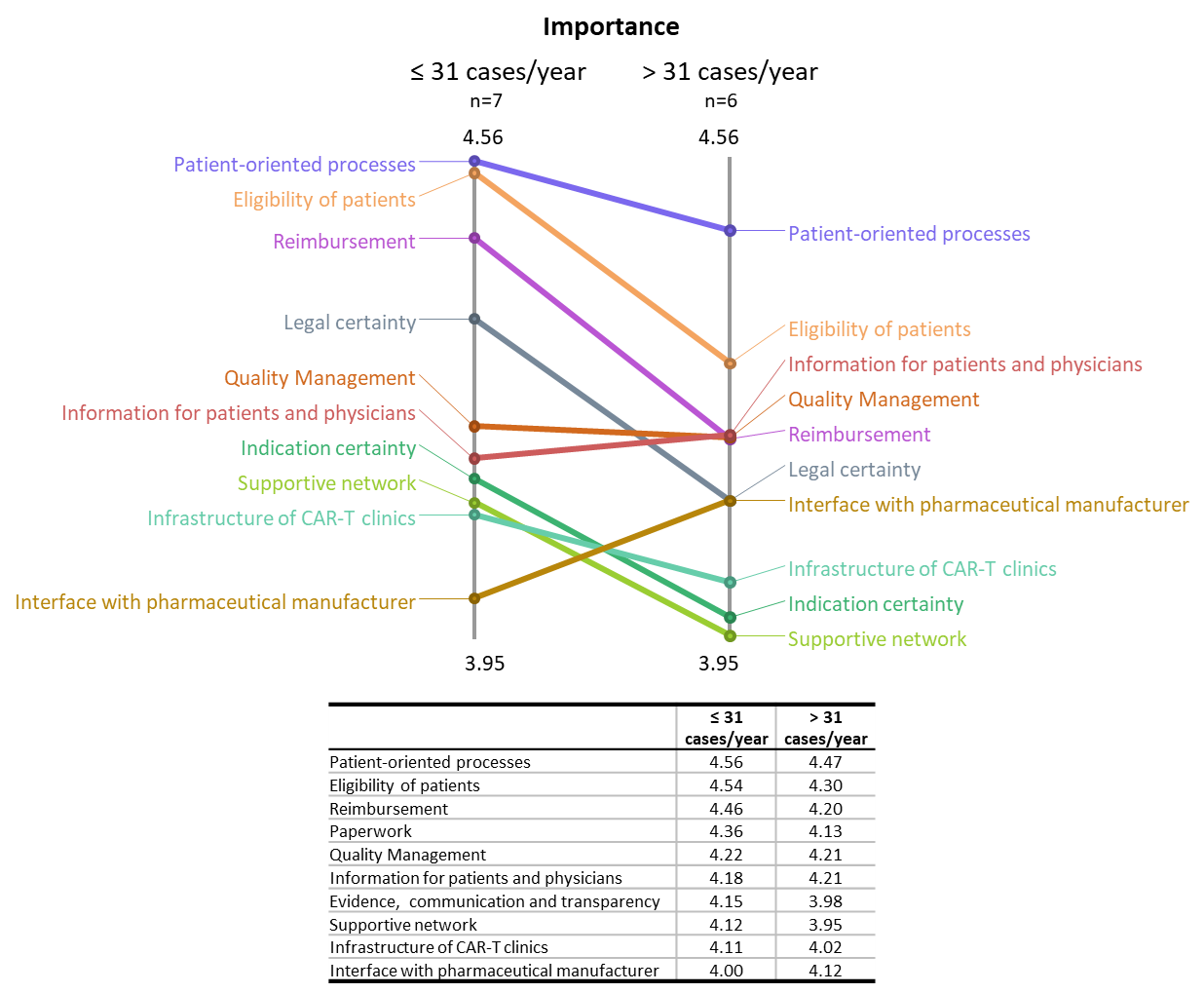 |
| --- |
| Average importance ratings per cluster per subgroup are contrasted. Axes values are *absolute*, i.e., *overall* minimum and maximum values (not per dimension) are reflected. Subgroups are defined based on the CAR-T cases that participants/their clinics are involved in per year (below or above the mean of 31 cases/year). 13 participants indicated a case number per year and completed the importance rating. Ratings were done on a 5-point Likert scale ranging from 1 to 5. |
